# Restriction of memory B cell differentiation at the germinal center B cell positive selection stage

**DOI:** 10.1084/jem.20191933

**Published:** 2020-05-14

**Authors:** Amparo Toboso-Navasa, Arief Gunawan, Giulia Morlino, Rinako Nakagawa, Andrea Taddei, Djamil Damry, Yash Patel, Probir Chakravarty, Martin Janz, George Kassiotis, Robert Brink, Martin Eilers, Dinis Pedro Calado

**Affiliations:** 1 Immunity and Cancer, Francis Crick Institute, London, UK; 2 Retroviral Immunology, Francis Crick Institute, London, UK; 3 Bioinformatics and Biostatistics, Francis Crick Institute, London, UK; 4 Experimental and Clinical Research Center, Max Delbrück Center for Molecular Medicine and Charité – Universitätsmedizin Berlin, Berlin, Germany; 5 Immunology Division, Garvan Institute of Medical Research, Darlinghurst, New South Wales, Australia; 6 Theodor Boveri Institute and Comprehensive Cancer Center Mainfranken, Biocenter, University of Würzburg, Würzburg, Germany; 7 Peter Gorer Department of Immunobiology, School of Immunology & Microbial Sciences, King’s College London, London, UK

## Abstract

Memory B cells (MBCs) are key for protection from reinfection. However, it is mechanistically unclear how germinal center (GC) B cells differentiate into MBCs. MYC is transiently induced in cells fated for GC expansion and plasma cell (PC) formation, so-called positively selected GC B cells. We found that these cells coexpressed MYC and MIZ1 (MYC-interacting zinc-finger protein 1 [ZBTB17]). MYC and MIZ1 are transcriptional activators; however, they form a transcriptional repressor complex that represses MIZ1 target genes. Mice lacking MYC–MIZ1 complexes displayed impaired cell cycle entry of positively selected GC B cells and reduced GC B cell expansion and PC formation. Notably, absence of MYC–MIZ1 complexes in positively selected GC B cells led to a gene expression profile alike that of MBCs and increased MBC differentiation. Thus, at the GC positive selection stage, MYC–MIZ1 complexes are required for effective GC expansion and PC formation and to restrict MBC differentiation. We propose that MYC and MIZ1 form a module that regulates GC B cell fate.

## Teaser

Germinal center (GC) positively selected B cells are primarily fated for GC expansion and plasma cell formation. This study reveals that memory B cell differentiation is restricted in positively selected B cells by the MYC–MIZ1 transcriptional repressor complex.

## Graphical Abstract

**Figure:**
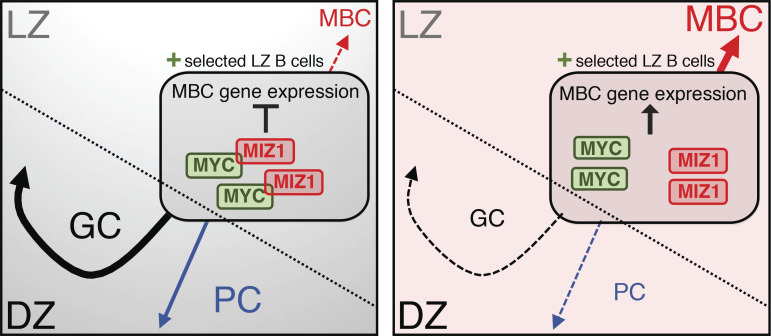

## Introduction

The germinal center (GC) is an antigen- and T cell–dependent reaction in which B cells undergo affinity maturation and differentiation (De Silva and Klein, 2015; Victora and Nussenzweig, 2012). In GCs, B cells cyclically migrate between an area called the dark zone (DZ), which is enriched for proliferating cells and where somatic hypermutation occurs, and an area called the light zone (LZ), in which B cells retrieve antigen from follicular (FO) dendritic cells (FDCs) through their B cell receptor (BCR) and present that antigen to T cells (Allen et al., 2004; Kepler and Perelson, 1993; Victora et al., 2010). T cell help, including CD40L-CD40 engagement, positively selects a fraction (∼5–20%) of LZ B cells, and our work and that of others showed that positive selection critically involves induction of MYC to license cell cycle, after which cells migrate back to the DZ, leading to GC expansion (Calado et al., 2012; Dominguez-Sola et al., 2012; Finkin et al., 2019; Luo et al., 2018; Schwickert et al., 2011). More recently, it was shown that positively selected LZ B cells (LZ MYC^+^ cells) are further composed of plasma cell (PC) precursors and that these also express *Myc* (Ise et al., 2018).

In addition to expansion in the GC and PC differentiation, LZ B cells also differentiate into memory B cells (MBCs). MBCs are key for long-term protection from reinfection, but how their fate is specified is poorly understood. MBC differentiation was thought to be an unregulated process (Inoue et al., 2018; Smith et al., 2000). Studies have shown, however, that MBCs have, in general, lower antigen affinity compared with LZ B cells fated for GC expansion and PC differentiation (De Silva and Klein, 2015; Shinnakasu et al., 2016; Weisel et al., 2016). Recently, it was found that LZ B cells expressing high levels of the gene encoding the transcription factor BACH2 are favored for MBC differentiation (Shinnakasu et al., 2016) and that quiescent LZ B cells are enriched for MBC precursors (Laidlaw et al., 2017; Suan et al., 2017; Wang et al., 2017). MYC is critically required for cell cycle entry of LZ MYC^+^ cells, and these cells are primarily fated for GC expansion and PC differentiation (Calado et al., 2012; Dominguez-Sola et al., 2012; Ise et al., 2018). We therefore raised the question whether MYC activity in LZ MYC^+^ cells restricts MBC differentiation.

In human cancers, MYC and the transcription activator MIZ1 (MYC-interacting zinc-finger protein 1 [ZBTB17]) can form a protein complex that represses the expression of MIZ1 target genes, most notably cyclin-dependent kinase inhibitor genes such as *CDKN1A* (Conacci-Sorrell et al., 2014; Peukert et al., 1997; Wiese et al., 2013). Mechanistically, MYC displaces MIZ1 coactivators EP300 and NPM1, converting MIZ1 from a transcriptional activator to a transcriptional repressor (Staller et al., 2001; Walz et al., 2014; Wanzel et al., 2008). Currently, the functions of MYC–MIZ1 complexes in physiology remain undetermined (Wiese et al., 2013). However, given that quiescent LZ B cells are enriched for MBC precursors (Laidlaw et al., 2017; Suan et al., 2017; Wang et al., 2017) and that MYC–MIZ1 complexes regulate cell cycle, we hypothesized that MYC–MIZ1 complex activity regulates MBC differentiation.

We found that at the positive selection stage GC B cells mostly coexpress MYC and MIZ1. The absence of MYC–MIZ1 complexes impaired cell cycle entry of LZ MYC^+^ cells, reducing GC expansion in a CDKN1A-independent manner, and interfered with PC formation. Notably, derepression of MIZ1 target genes led to a gene expression profile (GEP) alike that of MBCs, and mice lacking MYC–MIZ1 complexes had increased MBC differentiation. We propose that the transcription factors MYC and MIZ1 form a module that regulates the fate of positively selected GC B cells.

## Results

### Positively selected GC B cells mostly coexpress MYC and MIZ1

**Figure S1.**
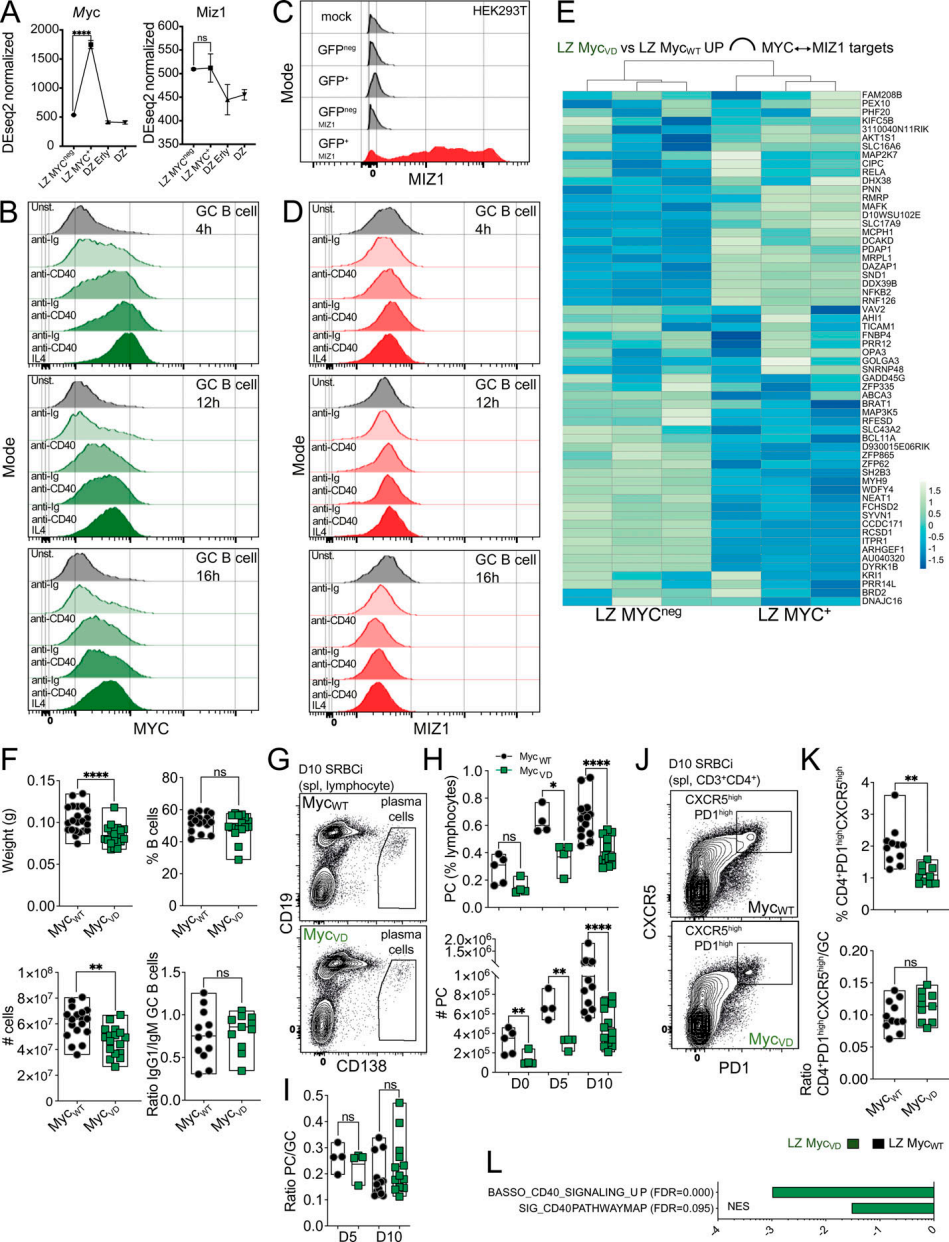
**Gene expression and cellular populations in the absence of MYC–MIZ1 complexes. (A)** RNA expression of *Myc* (left) and *Miz1* (right) in wild-type mice. DZ, DZ cells negative for Ap4; DZ Erly, DZ cells positive for Ap4; LZ MYC^neg^, LZ cells negative for MYC and AP4; LZ MYC^+^, LZ cells positive for MYC and AP4; (Chou et al., 2016). **(B)** Representative flow cytometry of intracellular staining for MYC in splenic GC B cells of wild-type mice isolated at day 10 after SRBC immunization (SRBCi) after 4 h (top), 12 h (middle), and 16 h (bottom) in vitro in media (Unst.) and in media in the presence of anti-Ig (anti-IgM + anti-IgG), anti-CD40, anti-Ig + anti-CD40, and anti-Ig + anti-CD40 + IL-4. **(C)** Testing of intracellular staining for MIZ1 in HEK293T nontransduced (mock) and transiently transduced with a plasmid expressing GFP (GFP^pos^ and GFP^neg^) or a plasmid expressing *MIZ1* and GFP as a bicistronic RNA (GFP^pos MIZ1^ and GFP^neg MIZ1^). **(D)** Representative flow cytometry of intracellular staining for MIZ1 as in B. **(E)** Heatmap representation of RNA expression in LZ MYC^neg^ and LZ MYC^+^ of wild-type mice for genes bound in their promoters by MIZ1 and MYC “MYC↔MIZ1” as determined by ChIP-seq in mouse B cells. **(F)** Top left: Weight of the spleen of Myc_WT_ and Myc_VD_ at day 10 after SRBC immunization. Top right: Fraction of splenic B lymphocytes of Myc_WT_ and Myc_VD_ at day 10 after SRBC immunization. Bottom left: Absolute cell number of splenic B cells of Myc_WT_ and Myc_VD_ at day 10 after SRBC immunization. Bottom right: Ratio of IgG1^+^ GC B cells over IgM^+^ GC B at day 10 after SRBC immunization. **(G)** Representative flow cytometry gating strategy of PCs in Myc_WT_ and Myc_VD_ at day 10 after SRBC immunization (SRBCi). spl, spleen. **(H)** Cumulative data for PCs, analyzed as in G at days 0, 5, and 10 after SRBC immunization. Top: Fraction of cells within lymphocytes. Bottom: Absolute cell number. **(I)** Ratio of PCs over GC B cells. **(J)** Representative flow cytometry gating strategy for splenic CD4^+^ CXCR5^+^ PD1^+^ T cells (that mostly contain Tfh cells) of Myc_WT_ and Myc_VD_ at day 10 after SRBC immunization. **(K)** Cumulative data for splenic CD4^+^ CXCR5^+^ PD1^+^ T cells. Top: Fraction within CD4^+^ T cells, analyzed as in J. Bottom, ratio between CD4^+^ CXCR5^+^ PD1^+^ T cells over GC B cells. **(L)** Bar graph displaying GSEA of gene signature “BASSO_CD40_SIGNALING_UP” and “SIG_CD40_PATHWAY” enrichment in the GEP of LZ B cells of Myc_WT_ and Myc_VD_. FDR, false discovery rate; NES, normalized enrichment score. Each symbol (F top left: Myc_WT_ *n* = 22, Myc_VD_ *n* = 20, F bottom left: Myc_WT_ *n* = 18, Myc_VD_ *n* = 17; F top right: Myc_WT_ *n* = 16, Myc_VD_ *n* = 16, F bottom right: Myc_WT_ *n* = 12, Myc_VD_ *n* = 10; H and I: day 0 Myc_WT_ *n* = 5, Myc_VD_ *n* = 4; day 5 Myc_WT_ *n* = 4, Myc_VD_ *n* = 4; day 10 Myc_WT_ *n* = 14, Myc_VD_ *n* = 13; K: Myc_WT_ *n* = 11, Myc_VD_ *n* = 10) represents an individual mouse; small horizontal lines show median, minimum, and maximum values. (A) ****, P ≤ 0.0001 (DEseq2). **(F–K)** *, P ≤ 0.05; **, P ≤ 0.01; ****, P ≤ 0.0001 (unpaired two-tailed Student’s *t* test). Data are representative of two (B–D, H, and I on days 0 and 5; and K) and three (F, H, and I on day 10) independent experiments. ns, not significant.

**Figure 1.**
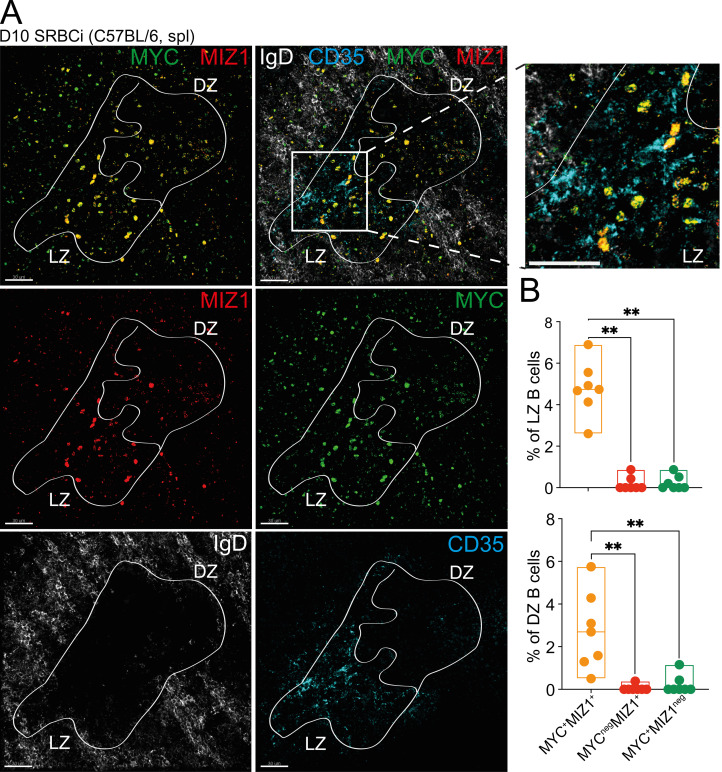
**Positively selected GC B cells mostly coexpress MYC and MIZ1. (A)** Representative confocal immunofluorescence of a splenic GC at day 10 (D10) after SRBC immunization (SRBCi) in a wild-type C57BL/6 mouse (MIZ1, red; MYC, green; IgD, white; and FDC/CD35, cyan). Lines delineate GC and LZ/DZ borders. Scale bars, 30 µm. **(B)** Quantification of the fraction of double-positive MIZ1^+^MYC^+^ and MIZ1^+^, MYC^+^ single-positive cells in the LZ (top) and DZ (bottom), stained as in A. Each symbol (B) represents an individual GC; small horizontal lines show median, minimum, and maximum values. Data in B are representative of three independent experiments (three mice, and approximately two GCs per mouse/per experiment). **, P ≤ 0.01 (unpaired two-tailed Student’s *t* test).

We first assessed the expression of *Miz1* in GC B cell subpopulations, including LZ MYC^+^ cells, using publicly available data (Chou et al., 2016). In contrast to *Myc*, which was strongly induced in LZ MYC^+^ cells, the expression of *Miz1* in these cells was similar to that of LZ B cells negative for MYC (LZ MYC^neg^ cells; Fig. S1 A). Next, we performed immunofluorescence of spleens of wild-type mice at 10 d after immunization with sheep RBCs (SRBCs) and delineated GCs (IgD^neg^), DZ and LZ (FDC depleted and enriched, respectively), and MYC expression to identify positively selected GC B cells. We found MIZ1 to be mostly coexpressed with MYC (Fig. 1, A and B). Collectively, these data indicated that MIZ1 expression is primarily regulated at a posttranscriptional level. MYC expression in GC B cells is synergistically induced by BCR and CD40 coengagement (Fig. S1 B; Luo et al., 2018). However, we found that BCR and CD40 coengagement was insufficient to induce MIZ1 expression in GC B cells stimulated in vitro (Fig. S1, C and D). Thus, although MYC and MIZ1 coexpression occurs in LZ MYC^+^ cells, the requirements for their induction are not identical.

### MIZ1 target genes are up-regulated in the absence of MYC–MIZ1 complexes

**Figure 2.**
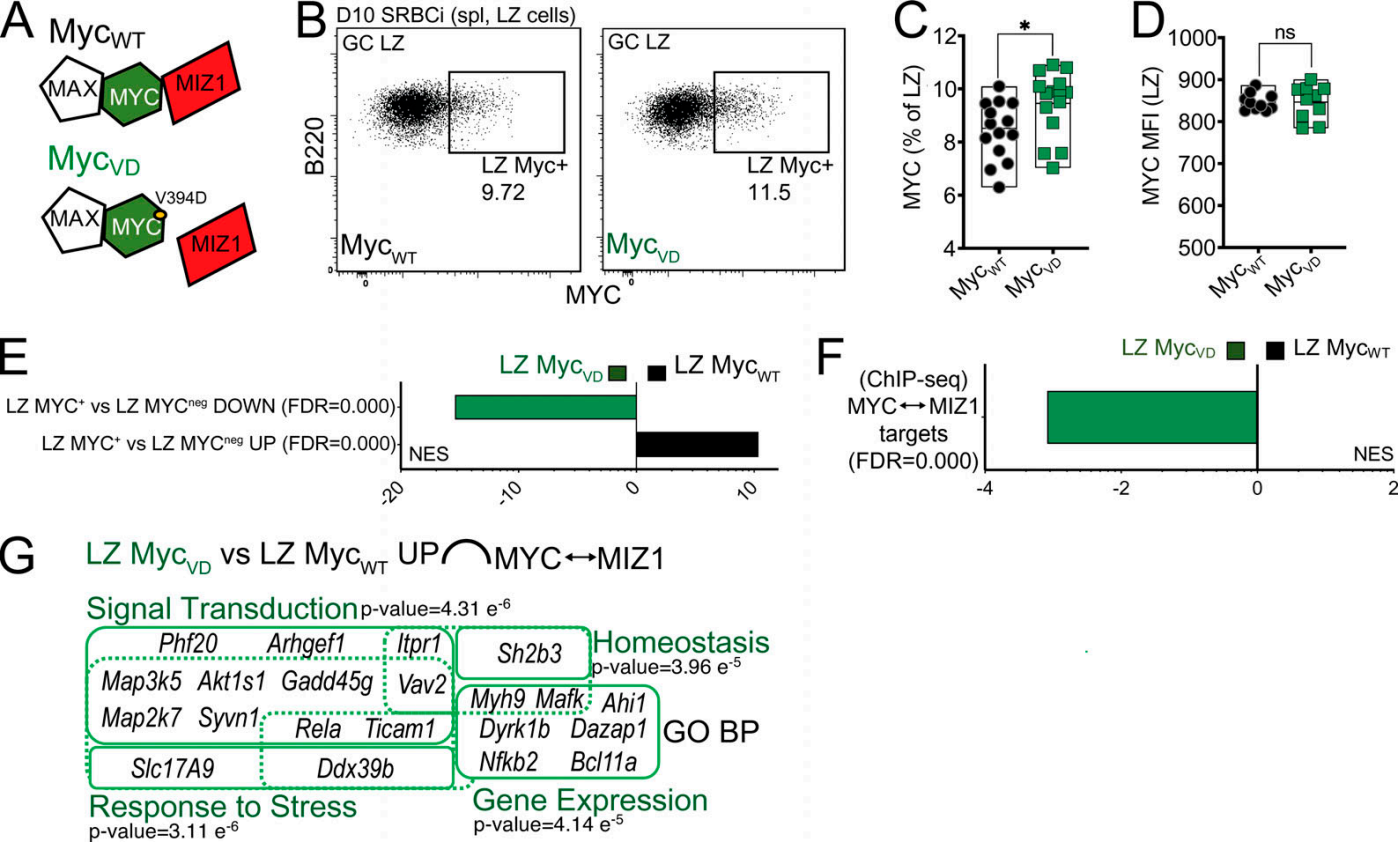
**MIZ1 target genes are up-regulated in the absence of MYC–MIZ1 complexes. (A)** Schematic representation of MYC–MIZ1 complexes. The V394D mutation in MYC abrogates the protein–protein interaction with MIZ1, but not with MAX. **(B)** Intracellular staining for MYC in splenic LZ GC B cells of Myc_WT_ and Myc_VD_ at day 10 after SRBC immunization (SRBCi). spl, spleen. **(C)** Cumulative data of the fraction of MYC^+^ cells in splenic LZ of Myc_WT_ and Myc_VD_, analyzed as in B. **(D)** Cumulative data of mean fluorescence intensity (MFI) in splenic LZ of Myc_WT_ and Myc_VD_, analyzed as in B. **(E)** Bar graph displaying GSEA of gene signature “LZ MYC^+^ vs. LZ MYC^neg^ DOWN” and “LZ MYC^+^ vs. LZ MYC^neg^ UP” enrichment in the GEP of LZ B cells of Myc_WT_ and Myc_VD_. FDR, false discovery rate; NES, normalized enrichment score. **(F)** Bar graph displaying normalized enrichment score of Myc_VD_ and Myc_WT_ LZ B cells GEP for genes bound in their promoters by MIZ1 and MYC “MIZ1↔MYC” as determined by ChIP-Seq in mouse B cells. **(G)** Graphical representation of enrichment of GO biological processes (GO_BP) within up-regulated genes in the LZ B cells of Myc_VD_ compared with Myc_WT_ with promoters bound by MIZ1 and MYC “MIZ1↔MYC”. Each symbol (C: Myc_WT_ *n* = 14, Myc_VD_ *n* = 14; D: Myc_WT_ *n* = 10, Myc_VD_ *n* = 10) represents an individual mouse; small horizontal lines are median, minimum, and maximum values. *, P ≤ 0.05 (unpaired two-tailed Student’s *t* test). Data in C and D are representative of three independent experiments. ns, not significant.

To investigate if MIZ1 target genes are regulated by MYC–MIZ1 complexes in positively selected GC B cells, we used a genetically modified mouse strain (Myc_VD_) that carries a MYC mutant encoded at the endogenous *Myc* locus (Herold et al., 2002; Saba et al., 2011). In these mice, replacement of a valine at position 394 by aspartic acid (V394D, Myc_VD_) abrogates MYC–MIZ1 interaction without interfering with the binding of MYC to its obligatory partner, MAX, and hence MYC transcriptional activation (Fig. 2 A; Herold et al., 2002; Saba et al., 2011; Walz et al., 2014). We first investigated the expression of MYC in Myc_VD_. We immunized Myc_VD_ and wild-type mice (Myc_WT_) with SRBC and determined 10 d later the fraction of LZ MYC^+^ cells using intracellular stain and flow cytometry. Myc_VD_ contained a slight but significantly increased fraction of LZ MYC^+^ cells compared with Myc_WT_ (Fig. 2, B and C), whereas MYC expression levels were identical between genotypes (Fig. 2 D). Thus, the absence of MYC–MIZ1 complexes did not impair the induction of MYC nor its expression level.

To investigate if the absence of MYC–MIZ1 complexes altered gene expression we FACS-purified LZ B cells of Myc_VD_ and Myc_WT_ and performed RNA sequencing (RNA-seq) followed by bioinformatic analysis. We first asked whether the GEP of LZ MYC^+^ cells was altered in the absence of MYC–MIZ1 complexes. For that, we used publicly available RNA-seq datasets (Chou et al., 2016) and generated signatures of genes down-regulated (*LZ MYC^+^* vs. *LZ MYC^neg^ DOWN*) or up-regulated (*LZ MYC^+^* vs. *LZ MYC^neg^ UP*) in LZ MYC^+^ compared with LZ MYC^neg^ cells. Using gene set enrichment analysis (GSEA), we found that the Myc_VD_ LZ GEP was significantly enriched for the “*LZ MYC^+^* vs. *LZ MYC^neg^ DOWN*” gene signature and that the Myc_WT_ LZ GEP was significantly enriched for the “*LZ MYC^+^* vs. *LZ MYC^neg^ UP*” gene signature (Fig. 2 E). These data indicated that the absence of MYC–MIZ1 complexes in LZ MYC^+^ cells profoundly altered their GEP, possibly due to the up-regulation of MIZ1 target genes repressed by MYC–MIZ1 complexes in those cells.

To identify differentially expressed genes between LZ Myc_VD_ and LZ Myc_WT_ that are direct targets of MYC–MIZ1 complexes, we generated and analyzed MYC and MIZ1 chromatin immunoprecipitation sequencing (ChIP-seq) in mouse B cells. In agreement with the absence of the repressive activity of MYC–MIZ1 complexes, the LZ Myc_VD_ GEP was significantly enriched for the expression of MIZ1 target genes that are bound by MYC compared with Myc_WT_ (Fig. 2 F). We found 60 MIZ1 target genes bound by MYC that were significantly up-regulated in the LZ of Myc_VD_ compared with that of Myc_WT_ (Fig. S1 E). A fraction of these genes (21 genes) enriched for the Gene Ontology (GO) biological processes related to signal transduction, response to stress, homeostasis, and gene expression (Fig. 2 G). These included genes encoding known tumor suppressors, namely *Arhgef1*, a RhoA-specific guanine nucleotide exchange factor frequently lost in GC B cell–derived lymphomas (Muppidi et al., 2014); the cytokine-regulated *Gadd45g*, required in hematopoietic stem cell differentiation and lineage selection (Lu et al., 2001; Thalheimer et al., 2014); and the U3 ubiquitin ligase *Sh2b3* that regulates JAK2 stability, which is frequently lost in acute lymphoblastic leukemia (Lv et al., 2017; Perez-Garcia et al., 2013). Genes involved in signal transduction included *Itpr1*, a Ca^2+^ channel required for normal B cell development and function (Tang et al., 2017); *Map2k7*, which directly activates c-JUN and is known to have an antiproliferative activity in B cells (Sasaki et al., 2001; Tournier et al., 1997); the AKT substrate *Akt1s1*, which negatively regulates mTOR activity (Sancak et al., 2007); and *Vav2*, which is critical for humoral immune responses and B cell maturation (Doody et al., 2001). Genes involved in gene expression included *Myh9*, which regulates BCR-mediated antigen acquisition and B cell activation (Hoogeboom et al., 2018); and multiple transcription factors, including *Bcl11a*, which is essential for B cell development and repressed by BLIMP1 during PC differentiation (Minnich et al., 2016; Yu et al., 2012); *Rela*, which was shown to be required for GC-derived PC formation and more recently also for MBC differentiation (Heise et al., 2014; Koike et al., 2019); and *Mafk*, which encodes for the transcription factor MAFK with which BACH2 heterodimerizes to bind target genes in B cells (Huang et al., 2014; Oyake et al., 1996). In summary, we found increased expression of multiple MIZ1 target genes in the absence of MYC–MIZ1 complexes. These data, together with the described expression pattern of MYC and MIZ1 (Fig. 1), supported a role for MYC–MIZ1 complexes in LZ MYC^+^ cells.

### MYC–MIZ1 complexes are required for cell cycle entry of LZ B cells

**Figure 3.**
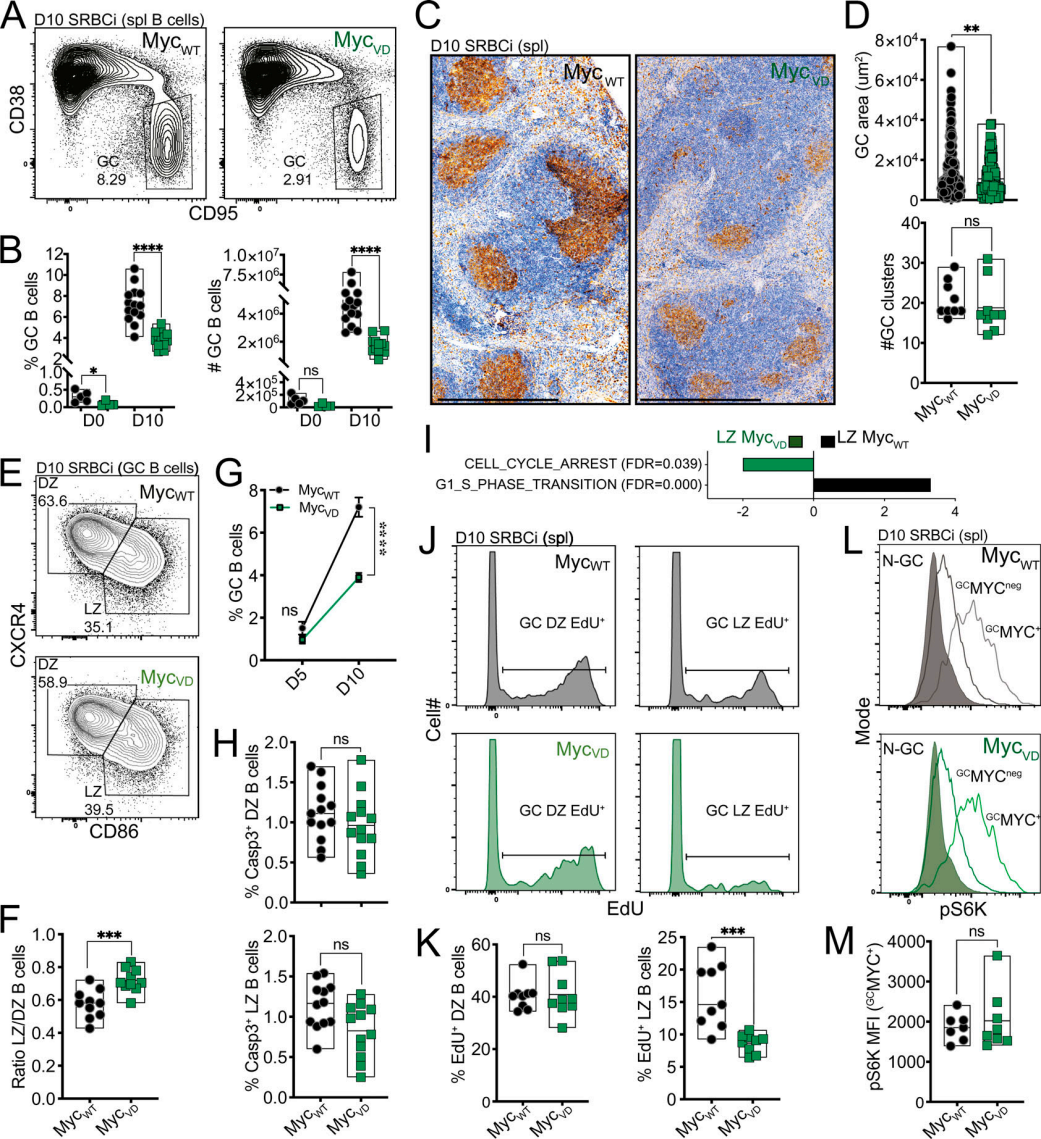
**MYC–MIZ1 complexes are required for cell cycle entry of LZ B cells.** **(A)** Representative flow cytometry of splenic GC B cells of Myc_WT_ and Myc_VD_ at day 10 after SRBC immunization (SRBCi). spl, spleen. **(B)** Cumulative data of Myc_WT_ and Myc_VD_ analyzed as in A before (day 0) and at day 10 after SRBC immunization. Left: Fraction of GC B cells within B cells. Right: Absolute cell number of GC B cells. **(C)** Representative histology of Myc_WT_ and Myc_VD_ at day 10 after SRBC immunization. PNA is used as GC marker, counterstained with hematoxylin. Scale bars, 500 µm. spl, spleen. **(D)** Top: Cumulative data of the area of each GC of Myc_WT_ and Myc_VD_, analyzed as in C. Bottom: Number of GC foci per spleen section analyzed as in C. **(E)** Representative flow cytometry of splenic DZ and LZ distribution within GC B cells at day 10 after SRBC immunization. **(F)** Cumulative data analyzed as in E and presented as LZ/DZ ratio. **(G)** Kinetics of the GC reaction and cumulative data of FACS analyses of splenic GC B cells of Myc_WT_ and Myc_VD_ at day 5 and day 10 after SRBC immunization, gated as in A. **(H)** Cumulative data of active caspase 3^+^ GC B cells within DZ (top) or LZ (bottom) of Myc_WT_ and Myc_VD_ at day 10 after SRBC immunization. **(I)** Bar graph displaying GSEA of gene signature “CELL_CYCLE_ARREST” and “G1_S_PHASE_TRANSITION” enrichment in the GEP of LZ B cells of Myc_WT_ and Myc_VD_. FDR, false discovery rate; NES, normalized enrichment score. **(J)** Flow cytometry of Myc_WT_ and Myc_VD_ at day 10 after SRBC immunization for the analysis of EdU incorporation in the DZ (left) and LZ (right). **(K)** Cumulative data for EdU incorporation in DZ (left) and LZ (right). Analyzed as in J. **(L)** Intracellular staining for phospho-S6 kinase (pS6K) in splenic non-GC (N-GC) B cells and MYC^+^, MYC^neg^ GC B cells (^GC^MYC^+^ and ^GC^MYC^neg^, respectively) of Myc_WT_ and Myc_VD_ at day 10 after SRBC immunization. **(M)** Cumulative data of phospho-S6 kinase mean fluorescence intensity (MFI) in MYC^+^ GC B cells (^GC^MYC^+^) splenic LZ of Myc_WT_ and Myc_VD_, analyzed as in L. Each symbol (B: day 0 Myc_WT_ *n* = 5, Myc_VD_ *n* = 5; day 10 Myc_WT_ *n* = 14, Myc_VD_ *n* = 14; F: Myc_WT_ *n* = 10, Myc_VD_ *n* = 10; H Myc_WT_ *n* = 12, Myc_VD_ *n* = 12; K: Myc_WT_ *n* = 9, Myc_VD_ *n* = 9; M: Myc_WT_ *n* = 7, Myc_VD_ *n* = 7) represents an individual mouse; small horizontal lines show median, minimum, and maximum values. Each symbol represents an individual GC (D) or mean and SEM (G). **, P ≤ 0.01; ***, P ≤ 0.001; ****, P ≤ 0.0001 (unpaired two-tailed Student’s *t* test). Data are representative of two (B: days 0; G: day 5; and M) or three (B: day 10; D, F, H, and K) independent experiments. ns, not significant.

We next tested whether MYC–MIZ1 complexes have a role in the GC reaction at the cell population level. Myc_VD_ had smaller spleens compared with Myc_WT_; however, the proportion of mature B cells was identical between genotypes (Fig. S1 F). 10 d after SRBC immunization, we observed a significant reduction in the fraction and number of GC B cells in Myc_VD_ compared with Myc_WT_ (Fig. 3, A and B). Histological analysis showed a significant reduction in the size of GC clusters in Myc_VD_ compared with Myc_WT_ (Fig. 3, C and D). However, the number of GC foci was identical between genotypes (Fig. 3 D). We also observed a small but significant increase in the LZ/DZ proportion in Myc_VD_ compared with Myc_WT_ (Fig. 3, E and F), whereas the proportion of IgG1- and IgM-expressing GC B cells was similar between genotypes (Fig. S1 F). These data showed that MYC–MIZ1 complexes were required for GC expansion, but not GC formation. In agreement, analysis of an earlier time-point after SRBC immunization (day 5), when GC clusters are formed but before massive expansion (Calado et al., 2012), Myc_VD_ and Myc_WT_ displayed a similar fraction of GC B cells (Fig. 3 G). In accordance with the knowledge that LZ MYC^+^ cells contain PC precursors we found a reduced fraction and number of PCs in Myc_VD_, which was proportional to the reduction observed for GC B cells (Fig. S1, G–I). These phenotypes did not seem to arise because of impaired T cell help. First, MYC expression levels, which are in part regulated by CD40 signaling (Luo et al., 2018), were similar between genotypes (Fig. 2, B–D). Second, the number of PD1^high^ CXCR5^high^ CD4^+^ T cells, which mostly represents GC T FO helper cells (Tfh cells), was proportional to that of GC B cells in Myc_VD_ and Myc_WT_ (Fig. S1, J and K). And third, genes up-regulated by CD40 signaling were found to be enriched in the Myc_VD_ LZ GEP compared with Myc_WT_ (Fig. S1 L).

**Figure S2.**
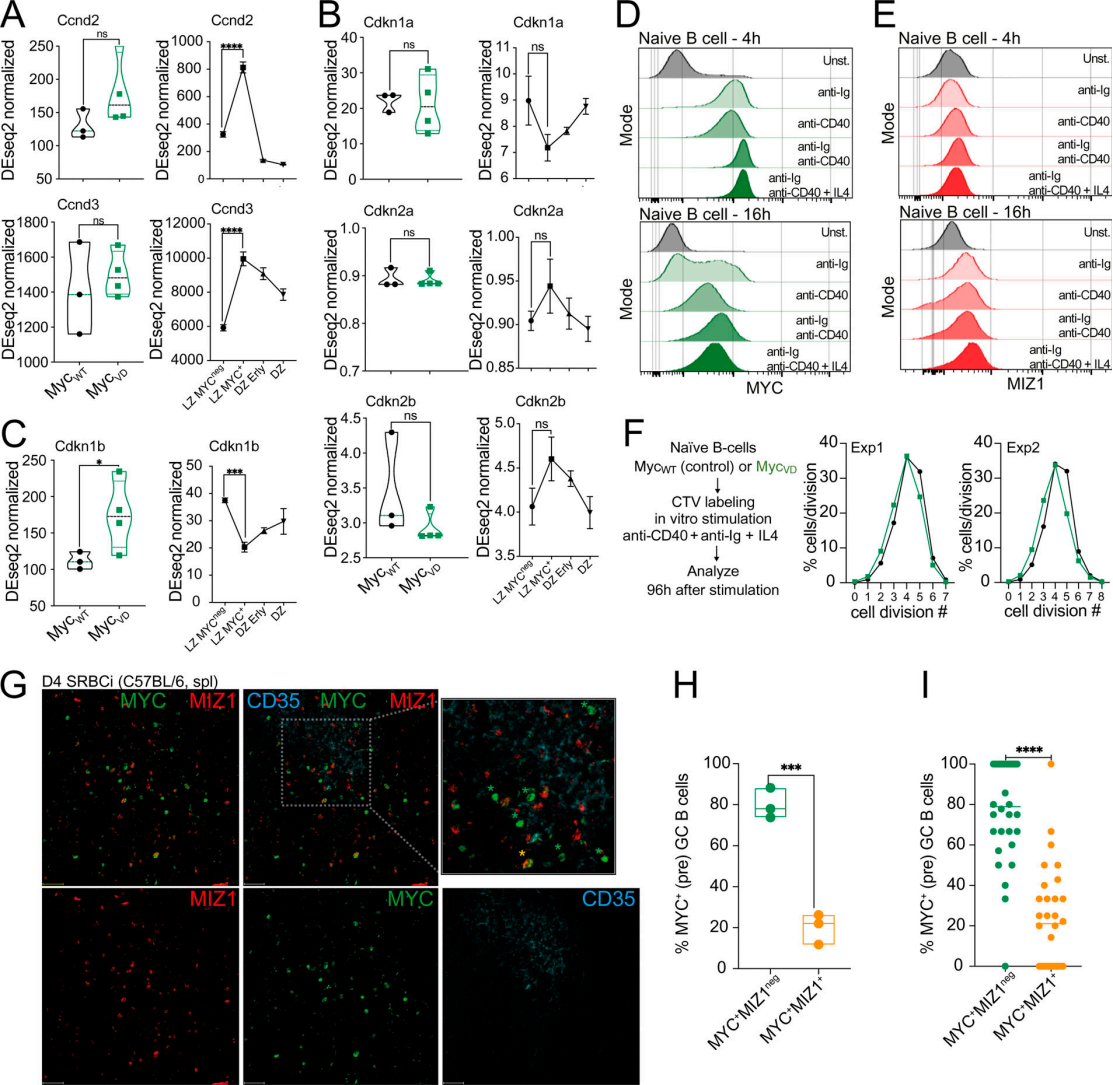
**Cell cycle genes and proliferation in the absence of MYC–MIZ1 complexes. (A)** Left: RNA expression of *Ccnd2* (top) and *Ccnd3* (bottom) derived from RNA-seq analysis of FACS purified Myc_VD_ and Myc_WT_ LZ B cells at day 10 after SRBC immunization. Right: RNA expression of *Ccnd2* (top) and *Ccnd3* (bottom) in wild-type mice. DZ, DZ cells negative for Ap4; DZ Erly, DZ cells positive for Ap4; LZ MYC^neg^, LZ cells negative for MYC and AP4; LZ MYC^+^, LZ cells positive for MYC and AP4 (Chou et al., 2016). **(B)** Left: RNA expression of CDK inhibitor genes *Cdkn1a* (top), *Cdkn2a* (middle), and *Cdkn2b* (bottom) derived as in A. Right: RNA expression of CDK inhibitor genes *Cdkn1a* (top), *Cdkn2a* (middle), and *Cdkn2b* (bottom) in wild-type mice as described in A. **(C)** Left: RNA expression of *Cdkn1b* (top) derived as in A. Right: RNA expression of *Cdkn1b* (top) in wild-type mice as described in A. **(D)** Representative flow cytometry of intracellular staining for MYC in splenic naive B cells of wild-type mice after 4 h (top) and 16 h (bottom) in vitro in media (Unst.) and in media with the presence of anti-Ig (anti-IgM + anti-IgG), anti-CD40, anti-Ig + anti-CD40, and anti-Ig + anti-CD40 + IL4. **(E)** Representative flow cytometry of intracellular staining for MIZ1 as in D. **(F)** Analysis of CellTrace Violet (CTV) dilution as a proxy of cell division of Myc_WT_ and Myc_VD_ naive B cells cultured for 96 h in vitro in media in the presence of anti-Ig + anti-CD40 + IL4. Two of three experiments are shown. **(G)** Representative confocal immunofluorescence of an FDC area at day 4 after SRBC immunization (SRBCi) in a wild-type C57BL/6 mouse (MIZ1, red; MYC, green; and FDC/CD35, cyan). White lines delineate the center of the FDC area. Scale bar, 20 µm. Green stars identify MYC^+^MIZ1^neg^ cells and orange start identifies a MIZ1^+^MYC^+^ cell. **(H)** Quantification of the fraction of MYC single positive (MYC^+^MIZ1^neg^) and MYC, MIZ1 double positive (MIZ1^+^MYC^+^) cells within MYC positive (pre) GC B cells in FDC areas of spleen of C57BL/6 mice at day 4 after SRBC immunization (D4 SRBCi). Each dot represents a mouse. **(I)** Analysis as in G. Each dot represents an FDC area. Each symbol (A–C: Myc_WT_ *n* = 3, Myc_VD_ *n* = 4) represents an individual mouse; small horizontal lines show median, minimum, and maximum values. **(A–C)** ***, P ≤ 0.001; ****, P ≤ 0.0001 (DEseq2). **(H and I)** *, P ≤ 0.05; ***, P ≤ 0.001; ****, P ≤ 0.0001 (unpaired two-tailed Student’s *t* test). Data are representative of two (D and E) and three (F, H, and I) independent experiments. ns, not significant.

**Figure 4.**
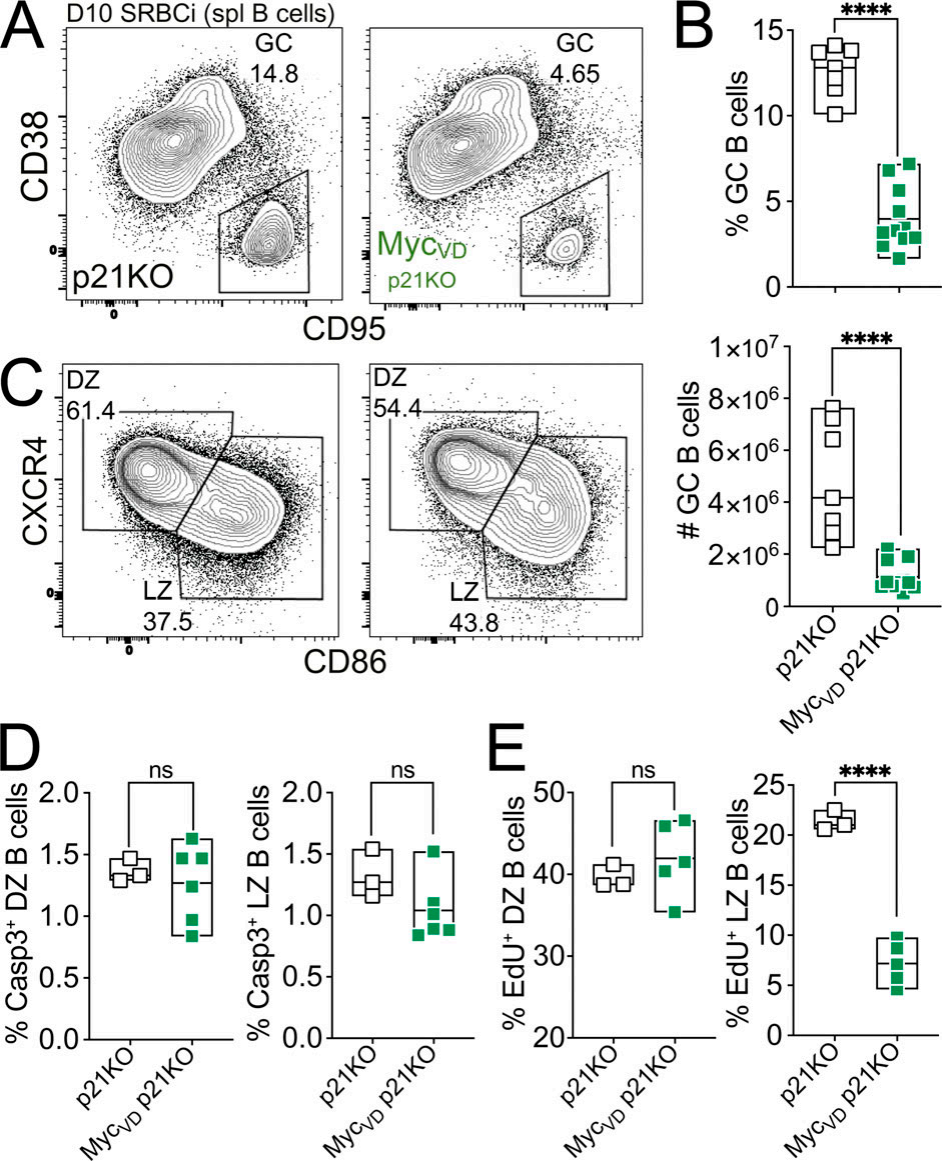
***Cdkn1a* ablation does not rescue cell cycle entry of LZ B cells in the absence of MYC–MIZ1 complexes. (A)** Representative flow cytometry of splenic GC B cells of p21KO (*Cdkn1a* KO) and Myc_VD_ p21KO mice at day 10 after SRBC immunization (SRBCi). spl, spleen. **(B)** Cumulative data of the fraction (top) and absolute cell number (bottom) of splenic GC B cells of p21KO and Myc_VD_ p21KO. Analyzed as in A. **(C)** Representative flow cytometry of splenic DZ and LZ B cells of p21KO and of Myc_VD_ p21KO at day 10 after SRBC immunization. **(D)** Cumulative data of active caspase 3^+^ splenic GC B cells in DZ (left) and LZ (right) of p21KO and Myc_VD_ p21KO at day 10 after SRBC immunization. **(E)** Cumulative data for EdU incorporation in DZ (left) and LZ (right) of p21KO and Myc_VD_ p21KO at day 10 after SRBC immunization. Each symbol (B: p21KO *n* = 7, Myc_VD_ p21KO *n* = 11; D: p21KO *n* = 3, Myc_VD_ p21KO *n* = 6; and E: p21KO *n* = 3, Myc_VD_ p21KO *n* = 5) represents an individual mouse; small horizontal lines show median, minimum, and maximum values. ****, P ≤ 0.0001 (unpaired two-tailed Student’s *t* test). Data are representative of two (B, D, and H) independent experiments. ns, not significant.

We next tested whether absence of MYC–MIZ1 complexes had an impact on the survival of GC B cells. However, we did not observe statistically significant differences in apoptosis between genotypes, although a trend toward a reduced fraction of active caspase 3^+^ cells was noticeable in the LZ of Myc_VD_ compared with Myc_WT_ (Fig. 3 H). Suggesting an altered cell cycle profile, GSEA of the GO term “CELL_CYCLE_ARREST” gene signature revealed a significant enrichment in the Myc_VD_ LZ GEP compared with Myc_WT_, whereas, in contrast, the GO term “G1-S_PHASE_TRANSITION” gene signature was enriched in Myc_WT_ LZ GEP compared with Myc_VD_ (Fig. 3 I). To determine whether the activity of MYC–MIZ1 complexes was required for the cell cycle of GC B cells, we performed 5-ethynyl-2′-deoxyuridine (EdU) pulse experiments. We found impaired cell cycle engagement of Myc_VD_ LZ B cells compared with Myc_WT_, whereas the fraction of DZ B cells engaged in cell cycle was identical between genotypes (Fig. 3, J and K). The cell cycle defect was not due to altered mTORC1 activity (Ersching et al., 2017), as LZ MYC^+^ cells of Myc_VD_ and Myc_WT_ displayed similar levels of phospho-S6 kinase (Fig. 3, L and M). We also did not observe altered expression of cyclin genes *Ccnd2* (a target of MYC; Bouchard et al., 1999; Calado et al., 2012) and *Ccnd3* in the absence of MYC–MIZ1 complexes (Cato et al., 2011; Peled et al., 2010; Fig. S2 A). Notably, the expression of the MIZ1 target genes *Cdkn1a*, *Cdkn2a*, and *Cdkn2b*, known to be repressed by MYC–MIZ1 complexes in cancer cells (Walz et al., 2014), were also similar between the LZ of Myc_VD_ and Myc_WT_ (Fig. S2 B). In agreement, ablation of *Cdkn1a* was insufficient to rescue the Myc_VD_ phenotype (Fig. 4, A–D), including the cell cycle defect of LZ B cells (Fig. 4 E). The expression of *Cdkn1b* was, however, increased in the LZ of Myc_VD_ compared with that of Myc_WT_ (Fig. S2 C), but neither we (Fig. S1 E) nor others (Walz et al., 2014) identified *Cdkn1b* as a direct MIZ1 target.

We next asked if the absence of MYC–MIZ1 complexes similarly altered the cell cycle of non-GC B cells in vitro. We first tested whether stimulation of naive B cells, including BCR and CD40 coengagement, induced MIZ1. Whereas MYC expression was already increased at 4 h after stimuli (Luo et al., 2018), MIZ1 expression was only increased after 16 h (Fig. S2, D and E). The absence of MYC–MIZ1 complexes did not significantly impact cell proliferation of in vitro–activated naive cells (Fig. S2 F). We next determined the expression of MYC and MIZ1 at the very early stages of the (pre-)GC reaction. Contrary to positively selected B cells in mature GCs, coexpression of MYC and MIZ1 was seldom observed in (pre-)GC B cells (Fig. S2, G–I). Overall these data indicated that the requirements for MIZ1 induction varied according to B cell stage and that MYC–MIZ1 complexes played a function in GC B cell expansion rather than formation. Specifically, in LZ MYC^+^ cells the absence of MYC–MIZ1 complexes dissociated MYC expression from cell cycle engagement.

### MIZ1 target genes are enriched in MBCs

**Figure 5.**
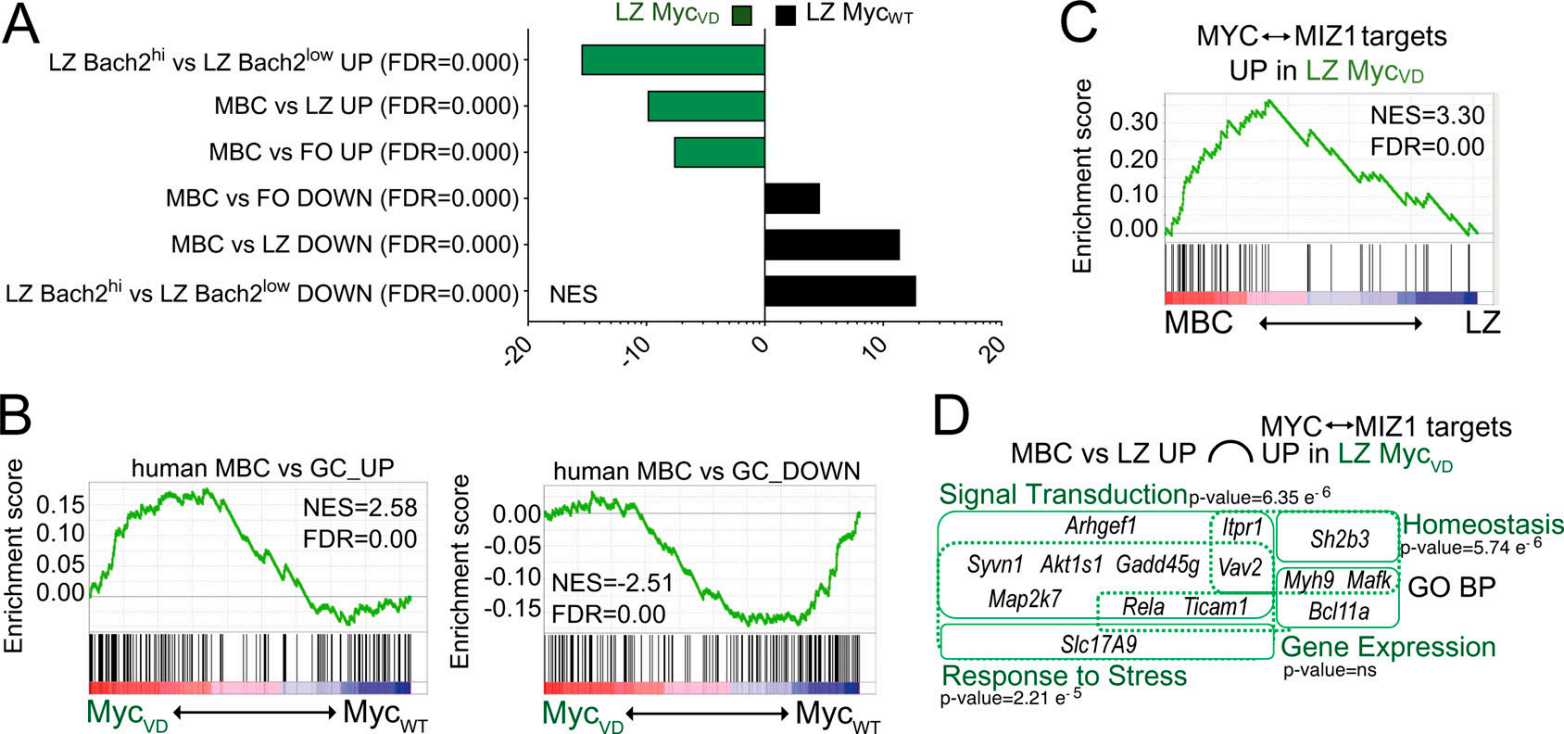
**MIZ1 target genes are enriched in MBCs. (A)** Bar graph displaying GSEA of gene signature “LZ Bach2^hi^ vs. LZ Bach2^low^ UP” and “LZ Bach2^hi^ vs. LZ Bach2^low^ DOWN”; “MBC vs. LZ UP” and “MBC vs. LZ DOWN”; “MBC vs. FO UP” and “MBC vs. FO DOWN” enrichment in the GEP of LZ B cells of Myc_WT_ and Myc_VD_. FDR, false discovery rate; NES, normalized enrichment score. **(B)** GSEA of GEP of LZ B cells of Myc_WT_ and Myc_VD_ for “(human) MBC vs. GC_UP” gene signature (left) and “(human) MBC vs. GC_DOWN” (right). **(C)** GSEA of GEP of MBC and LZ B cells using a gene signature composed of genes bound in their promoters by MIZ1 and MYC “MIZ1↔MYC” and up-regulated in LZ B cells of Myc_VD_ compared with that of Myc_WT_, as determined by ChIP-seq in mouse B cells. **(D)** Graphical representation of enrichment of GO biological processes (GO_BP) within up-regulated genes in LZ B cells of Myc_VD_ compared with Myc_WT_ that are targets of MIZ1 bound by MYC and enriched in MBCs.

**Figure S3.**
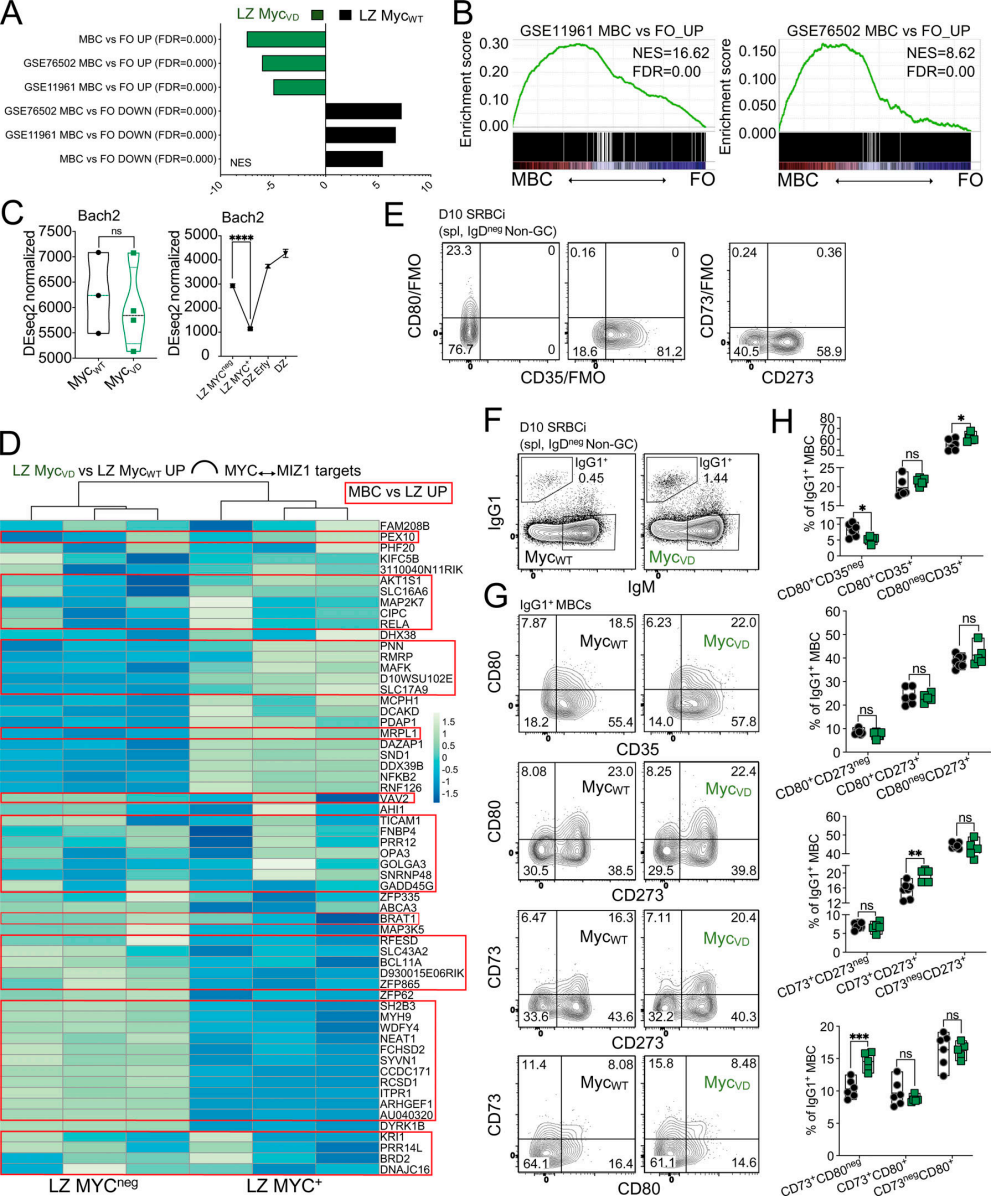
**MBC-associated genes and populations in mice lacking MYC–MIZ1 complexes. (A)** Bar graph displaying GSEA of GEP of LZ B cells of Myc_WT_ and Myc_VD_ for gene signature “MBC vs. FO UP”; “GSE76502 MBC vs. FO UP”; “GSE11961 MBC vs. FO UP”; “MBC vs. FO DOWN”; “GSE76502 MBC vs. FO DOWN”; “GSE11961 MBC vs. FO DOWN”. FDR, false discovery rate; NES, normalized enrichment score. **(B)** Left: GSEA of GEP of MBC and FO of this work for “GSE11961 MBC vs. FO_UP” gene signature. Right: GSEA of GEP of MBC and FO for “GSE76502 MBC vs. FO_UP” gene signature. **(C)** Left: RNA expression of *Bach2* in Myc_VD_ LZ B cells compared with Myc_WT_ LZ B cells, derived from RNA-seq analysis of FACS-purified cells at day 10 after SRBC immunization. Right: RNA expression of *Bach2*. DZ, DZ cells negative for AP4; DZ Erly, DZ cells positive for AP4; LZ MYC^neg^, LZ cells negative for both MYC and AP4; LZ MYC^+^, LZ cells positive for both MYC and AP4 (Chou et al., 2016). **(D)** Heatmap representation of RNA expression in LZ MYC^neg^ and LZ MYC^+^ for genes bound in their promoters by MIZ1 and MYC “MIZ1↔MYC” as determined by ChIP-seq in mouse B cells. Genes found significantly up-regulated in MBC vs. LZ B cells are presented within a red box. **(E)** Representative flow cytometry using Fluorescence Minus One (FMO) controls for CD35 (left), CD80 (middle), and CD73 (right). **(F)** Representative flow cytometry gating strategy for splenic IgG1^+^ MBCs of Myc_WT_ and Myc_VD_ at day 10 after SRBC immunization (SRBCi). spl, spleen. **(G)** Representative flow cytometry for surface marker staining of splenic IgG1^+^ MBCs of Myc_WT_ and Myc_VD_ at day 10 after SRBC immunization (SRBCi). spl, spleen. **(H)** Cumulative data for analysis as in G. Each symbol (C, left: Myc_WT_ *n* = 3, Myc_VD_ *n* = 4; H: Myc_WT_ *n* = 7, Myc_VD_ *n* = 5) represents an individual mouse, (C, left) small horizontal lines show median, minimum, and maximum values; (C, right) mean and SEM. **(C)** ****, P ≤ 0.0001 (DEseq2). **(H)** *, P ≤ 0.05; **, P ≤ 0.01; ***, P ≤ 0.001 (unpaired two-tailed Student’s *t* test). Data are representative of two (H) independent experiments. ns, not significant.

The impaired cell cycle engagement of LZ MYC^+^ cells of Myc_VD_ and the knowledge that MBC precursors are found within quiescent LZ B cells (Laidlaw et al., 2017; Suan et al., 2017; Wang et al., 2017) led us to investigated if the LZ Myc_VD_ GEP displayed a shift toward that of MBCs. We first generated an MBC GEP by performing RNA-seq of FACS-purified MBCs from day 10 SRBC-immunized wild-type mice. Using these data, we generated signatures of genes up-regulated (*MBC* vs. *LZ UP*) or down-regulated (*MBC* vs. *LZ DOWN*) in MBCs compared with LZ B cells. We found that the LZ Myc_VD_ GEP was significantly enriched for the “*MBC* vs. *LZ UP*” gene signature, whereas the LZ Myc_WT_ GEP was significantly enriched for the “*MBC* vs. *LZ DOWN*” gene signature (Fig. 5 A). To investigate whether this enrichment was the consequence of decreased cell cycle activity in the Myc_VD_ LZ we performed RNA-seq of FACS-purified FO B cells, known to be quiescent, and generated signatures of genes up-regulated (*MBC* vs. *FO UP*) or down-regulated (*MBC* vs. *FO DOWN*) in MBCs compared FO B cells. Similar to the previous analysis, we found that the Myc_VD_ LZ GEP was significantly enriched for the “*MBC* vs. *FO UP*” gene signature, whereas the LZ Myc_WT_ GEP was significantly enriched for the “*MBC* vs. *FO DOWN*” gene signature (Fig. 5 A). Identical results were observed when analyzing “MBC vs. FO” signatures using GEP datasets generated by others (Fig. S3 A), in agreement with the significant overlap between the MBC GEP generated in this work and that of others (Fig. S3 B; Kaji et al., 2012; Weisel et al., 2016). We also found a significant enrichment in the Myc_VD_ LZ GEP for genes up-regulated in MBC vs. GC B cells of human origin (human MBC vs. GC_UP), whereas the Myc_WT_ LZ GEP was enriched for genes down-regulated in MBC vs. GC B cells (human MBC vs. GC_DOWN; Fig. 5 B; Luckey et al., 2006). To further validate our analysis, we used a publicly available RNA-seq dataset comparing LZ B cells expressing high *Bach2* levels (LZ Bach2^hi^) that are enriched for MBC precursors and LZ B cells expressing low *Bach2* levels (LZ Bach2^low^) that are enriched for cells fated for GC expansion and PC differentiation (Shinnakasu et al., 2016). We found that the Myc_WT_ LZ GEP was significantly enriched for the gene signature “*LZ Bach2^hi^* vs. *LZ Bach2^low^ DOWN*” that is associated with GC expansion and PC fates (Fig. 5 A). In contrast, Myc_VD_ LZ GEP was significantly enriched for the gene signature “*LZ Bach2^hi^* vs. *LZ Bach2^low^ UP*” that is associated with MBC precursors (Fig. 5 A). In agreement with *Bach2* levels being down-regulated by T cell help (Shinnakasu et al., 2016), we found that the expression of *Bach2* was significantly reduced in LZ MYC^+^ compared with LZ MYC^neg^ cell subsets of wild-type mice (Fig. S3 C). However, *Bach2* levels were not different between LZ B cells of Myc_VD_ and Myc_WT_ (Fig. S3 D), further suggesting that CD40 signaling is not impaired in Myc_VD_ compared with Myc_WT_. Overall, these analyses indicate that the absence of MYC–MIZ1 complexes in LZ MYC^+^ cells altered the GEP of LZ B cells in part through the enrichment of genes expressed in MBCs.

Next, we investigated whether the identified 60 MIZ1 target genes bound by MYC and significantly up-regulated in the LZ of Myc_VD_ compared with that of Myc_WT_ (Fig. 2 G and Fig. S1 E) were enriched in the MBC GEP. 41 out of the 60 MIZ1 target genes (68%) had increased expression in MBCs vs. LZ B cells (Fig. 5, C and D), and the majority of these 41 genes (30/41, 73%) had reduced expression in LZ MYC^+^ compared with LZ MYC^neg^ cells (Fig. S3 D). These data reflected the repressive activity of MYC–MIZ1 complexes in LZ MYC^+^ cells and that these complexes directly repressed genes enriched in MBCs.

### MYC–MIZ1 complexes restrict MBC differentiation

**Figure 6.**
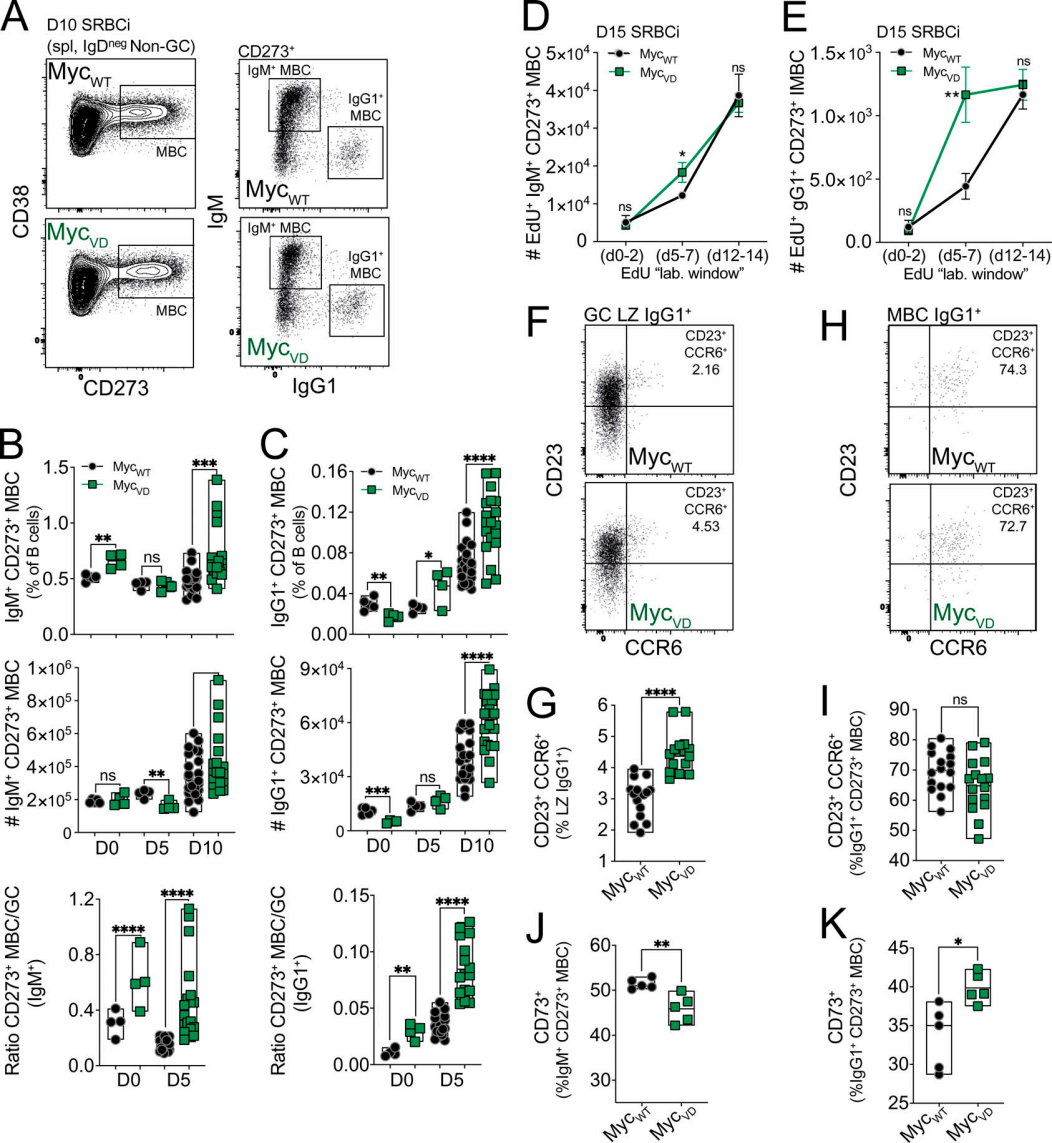
**MYC–MIZ1 complexes restrict MBC differentiation. (A)** Representative flow cytometry gating strategy for splenic IgM^+^ and IgG1^+^ MBCs (B220^+^ CD19^+^ CD38^high^ IgD^low^ CD273^+^) of Myc_WT_ and Myc_VD_ at day 10 after SRBC immunization (SRBCi). spl, spleen. **(B)** Cumulative data for splenic IgM^+^ CD273^+^ MBCs analyzed as in A at days 0, 5, and 10 after SRBC immunization. Top: Fraction of cells within B cells. Middle: Absolute cell number. Bottom: Ratio of IgM^+^ CD273^+^ MBCs over IgM^+^ GC B cells. **(C)** Cumulative data for splenic IgG1^+^ CD273^+^ MBCs analyzed as in A at days 0, 5, and 10 after SRBC immunization. Top: Fraction of cells within B cells. Middle: Absolute cell number. Bottom: Ratio of IgG1^+^ CD273^+^ MBCs over IgG1^+^ GC B cells. **(D and E)** Kinetics of splenic IgM^+^ CD273^+^ (D) and IgG1^+^ CD273^+^ (E) MBC production determined by EdU pulse-chase experiments at the indicated labeling windows. Cumulative FACS analyses of splenic MBCs analyzed as in A at day 15 after SRBC immunization (SRBCi). **(F)** Representative flow cytometry of CD23 and CCR6 expression within splenic IgG1^+^ LZ B cells of Myc_WT_ and Myc_VD_ at day 10 after SRBC immunization. **(G)** Cumulative data for the fraction of CD23^+^ CCR6^+^ within splenic IgG1^+^ LZ B cells from Myc_WT_ and Myc_VD_, analyzed as in F. **(H)** Representative flow cytometry of CD23 and CCR6 expression within splenic IgG1^+^ CD273^+^ MBCs of Myc_WT_ and Myc_VD_ at day 10 after SRBC immunization. **(I)** Cumulative data for the fraction of CD23^+^ CCR6^+^ within splenic IgG1^+^ CD273^+^ MBCs from Myc_WT_ and Myc_VD_, analyzed as in H. **(J)** Cumulative data for the fraction of CD73^+^ cells within splenic IgM^+^ CD273^+^ MBCs analyzed as in A at day 10 after SRBC immunization. **(K)** Cumulative data for the fraction of CD73^+^ cells within splenic IgG1^+^ CD273^+^ MBCs analyzed as in A at day 10 after SRBC immunization. Each symbol (B and C: day 0 Myc_WT_ *n* = 5, Myc_VD_ *n* = 4; day 5 Myc_WT_ *n* = 4, Myc_VD_ *n* = 4; day 10 Myc_WT_ *n* = 22, Myc_VD_ *n* = 19; G and I: Myc_WT_ *n* = 15, Myc_VD_ *n* = 16; J and K: Myc_WT_ *n* = 5, Myc_VD_ *n* = 5) represents an individual mouse; small horizontal lines show median, minimum, and maximum values or mean and SEM (D and E). *, P ≤ 0.05; **, P ≤ 0.01; ***, P ≤ 0.001; ****, P ≤ 0.0001 (unpaired two-tailed Student’s *t* test). Data are representative of two (B and C at days 0 and 5; D and E) or three (B and C at day 10; G–K) independent experiments. ns, not significant.

To assess whether MBC formation was altered in the absence of MYC–MIZ1 complexes, we initially immunized Myc_VD_ and Myc_WT_ with SRBC (Fig. 6 A). The fraction of IgM^+^ CD273^+^ MBCs within B cells was significantly increased in Myc_VD_ compared with Myc_WT_ at day 10 after SRBC immunization, albeit the absolute cell number not being statistically different between genotypes (Fig. 6 B). Nevertheless, considering that Myc_VD_ had reduced GC B cell numbers compared with Myc_WT_, a significantly increased IgM^+^ CD273^+^ MBC to IgM^+^ GC B cell ratio was observed, indicating that Myc_VD_ produced more IgM^+^ CD273^+^ MBCs than Myc_WT_ (Fig. 6 B). The fraction of IgG1^+^ CD273^+^ MBCs within B cells was significantly increased at day 5 after immunization in Myc_VD_ compared with Myc_WT_, but only a trend in terms of absolute cell numbers was observed (Fig. 6 C). However, at day 10 after SRBC immunization, the difference was clear, as both the fraction within B cells and the absolute number of IgG1^+^ CD273^+^ MBCs were significantly increased in Myc_VD_ compared with Myc_WT_ (Fig. 6 C). We concluded that the absence of MYC–MIZ1 complexes in LZ MYC^+^ cells increased MBC differentiation.

It is acknowledged that MBCs can be generated by both GC-dependent and GC-independent paths (Blink et al., 2005; Chan et al., 2009; Good-Jacobson and Tarlinton, 2012; Inamine et al., 2005; Takemori et al., 2014; Toyama et al., 2002). We therefore investigated the kinetics of MBC differentiation in Myc_VD_ and Myc_WT_ by performing EdU pulse-chase experiments in which “3-d labeling windows” are generated through EdU injection at specific time points after immunization (Weisel et al., 2016). The labeling windows chosen with respect to the immunization time point were: (1) days 0–2, which encompass the earlier stage of the GC reaction; (2) days 5–7, which encompasses critical time points of GC expansion; and (3) days 12–14 for a later GC stage (Calado et al., 2012). In these experiments, analysis of mice at day 15 after SRBC immunization revealed a significantly increased number of EdU^+^ IgM^+^ CD273^+^ MBCs and to a greater degree for EdU^+^ IgG1^+^ CD273^+^ MBCs in Myc_VD_ compared with Myc_WT_ at the day 5–7 labeling window (Fig. 6, D and E). These data showed that the absence of MYC–MIZ1 complexes in LZ MYC^+^ cells originated a marked increase of MBC formation at critical time points of GC expansion. We also found that the fraction of IgG1^+^ LZ B cells of Myc_VD_ was significantly enriched for cells expressing the MBC precursor marker CCR6 (Suan et al., 2017) compared Myc_WT_ (Fig. 6, F and G), whereas the fraction of CCR6^+^ cells within IgG1^+^ CD273^+^ MBCs was identical between genotypes (Fig. 6, H and I). These data indicated that MBC precursors were significantly increased within IgG1^+^ LZ B cells of Myc_VD_ compared Myc_WT_. Previous work in which GC B cell formation was abrogated demonstrated that the expression of CD73 marks GC-derived MBCs, whereas MBCs lacking CD73 can be of either GC or non-GC origin (Anderson et al., 2007; Kaji et al., 2012; Taylor et al., 2012). We found that ∼45% of the IgM^+^ CD273^+^ MBCs generated in Myc_VD_ were CD73^+^ compared with ∼50% in Myc_WT_ (Fig. 6 J), whereas ∼40% of IgG1^+^ CD273^+^ MBCs in Myc_VD_ were CD73^+^ compared with ∼35% in Myc_WT_ (Fig. 6 K). These data indicated a similar origin of MBCs between genotypes, and we concluded that the increase in MBC differentiation observed in the absence of MYC–MIZ1 complexes occurred primarily through a GC-dependent path. We also investigated the distribution of known MBC surface marker combinations (Anderson et al., 2007; Tomayko et al., 2010) within IgG1^+^ MBCs in Myc_VD_ compared with Myc_WT_ (Fig. S3, E–H). We found significantly increased fractions of CD73^+^CD80^neg^ and CD80^neg^CD35^+^ MBCs in Myc_VD_, whereas a decreased fraction of CD80^+^CD35^neg^ was observed in these mice (Fig. S3, F–H). Expression of CD80 was previously associated with a higher number of somatic mutations in the IgH variable region, whereas that of CD35 with reduced mutational load (Anderson et al., 2007). These data therefore indicated that the enlarged IgG1^+^ MBC pool of Myc_VD_ is composed of cells with lower affinity toward the antigen, consistent with increased MBC differentiation at critical time points of GC expansion (Fig. 6 E), when GC antigen affinity is likely to be low.

### Reduced affinity of the MBC pool in the absence of MYC–MIZ1 complexes

**Figure 7.**
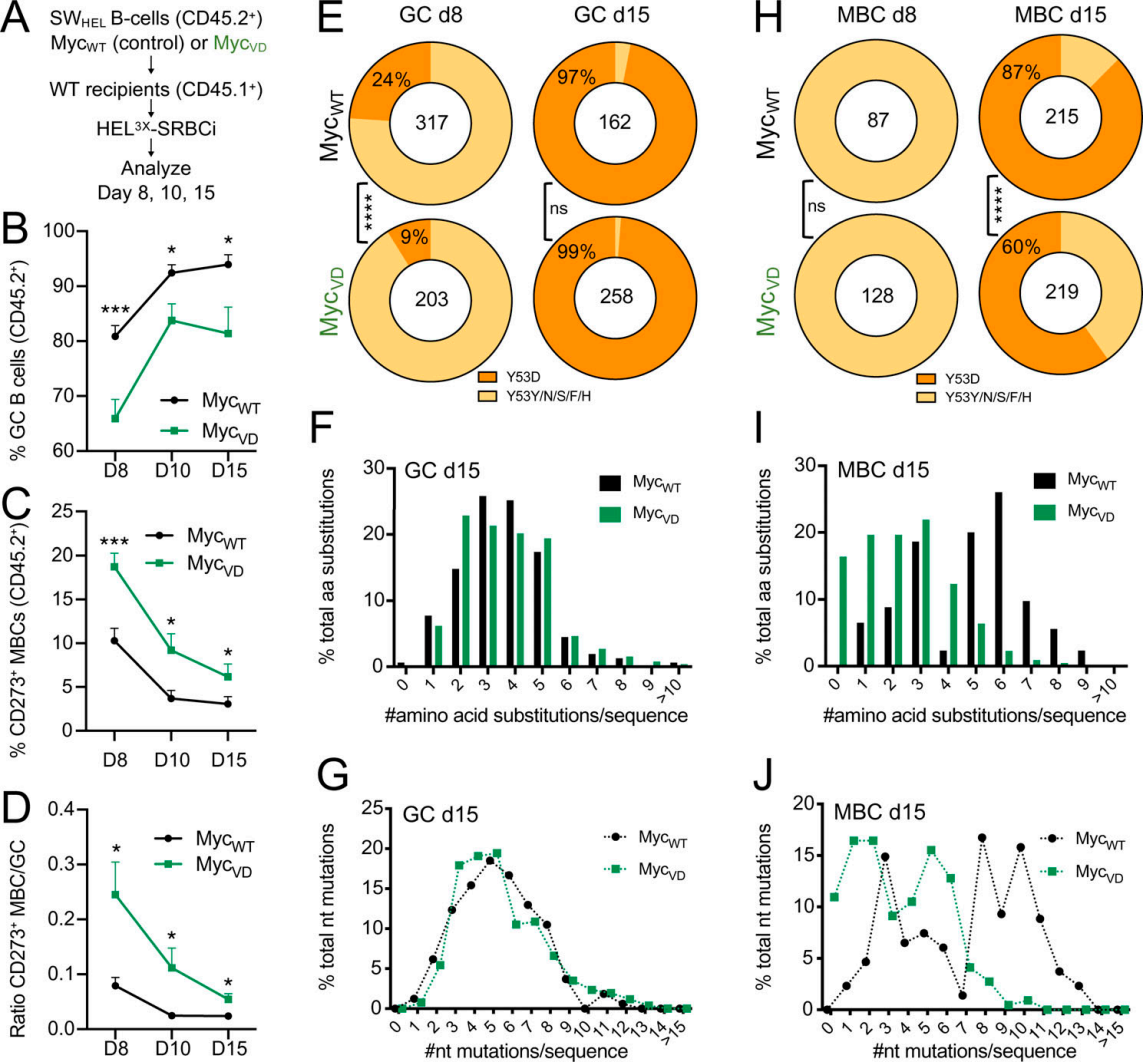
**Reduced affinity of the MBC pool in the absence of MYC–MIZ1 complexes. (A)** Experimental design. **(B)** Kinetics of GC B cells within splenic CD45.2^+^ donor cells at days 8, 10, and 15 after HEL^3X^ immunization. **(C)** Kinetics of CD273^+^ MBCs within splenic CD45.2^+^ donor cells at days 8, 10, and 15 after HEL^3X^ immunization. **(D)** Ratio of CD273^+^ MBCs to GC B cells at days 8, 10, and 15 after HEL^3X^ immunization. **(E)** Frequency of Y53D mutations within splenic IgG1^+^ GC B cells at day 8 (d8; left) and day 15 (right) after HEL^3X^ immunization; the number of analyzed IgH sequences is shown inside the circle. **(F)** Frequency of splenic IgG1^+^ GC B cells carrying the specified number of amino acid substitutions, analyzed at day 15 after HEL^3X^ immunization. **(G)** Frequency of nucleotide substitutions across the IgH V-region of SWHEL within splenic IgG1^+^ GC B cells at day 15 after HEL^3X^ immunization. **(H)** Frequency of Y53D mutations within splenic IgG1^+^ CD273^+^ MBCs at day 8 (left) and day 15 (right) after HEL^3X^ immunization, number of analyzed IgH sequences is shown inside the circle. **(I)** Frequency of splenic IgG1^+^ CD273^+^ MBCs cells carrying the specified number of amino acid substitutions, analyzed at day 15 after HEL^3X^ immunization. **(J)** Frequency of nucleotide substitutions across the IgH V-region of SWHEL within splenic IgG1^+^ CD273^+^ MBCs at day 15 after HEL^3X^ immunization. **(B–D)** Mean and SEM. *, P ≤ 0.05, **, P ≤ 0.01, ***, P ≤ 0.001 (unpaired two-tailed Student’s *t* test). Data are representative of three (B–D) independent experiments and cumulative analysis of five mice per genotype per time point (E–J). ns, not significant.

Following the analysis using SRBC immunization, we wanted to determine whether the absence of MYC–MIZ1 complexes impacted antigen affinity maturation. To study BCR antigen affinity, we generated compound mutant Myc_VD_ and Myc_WT_ carrying the SWHEL allelic system, in which B cells express a transgenic BCR recognizing hen egg lysozyme (HEL; Phan et al., 2003). In this system, the BCR affinity to HEL is very high (2 × 10^10^ M^−1^), but affinity maturation can be studied by immunizing mice with a mutant version of HEL called HEL^3X^, for which SWHEL BCR has much lower affinity (1.5 × 10^6^ M^−1^; Paus et al., 2006). According to established protocols (Paus et al., 2006), we purified B cells from Myc_VD_SWHEL and control Myc_WT_SWHEL and transferred them into congenic CD45.1 mice, after which recipient mice were immunized with HEL^3X^ (Fig. 7 A). We found at all time points of analysis (days 8, 10, and 15) a reduction in GC B cells and an increase of CD273^+^ MBCs within B cells derived from Myc_VD_SWHEL compared with Myc_WT_SWHEL (Fig. 7, B–D). These data reproduced the phenotypes observed in Myc_VD_ (Fig. 3 and Fig. 6) and demonstrated that the reduced GC expansion and increased MBC differentiation was due to the specific absence of MYC–MIZ1 complexes in B cells.

**Figure S4.**
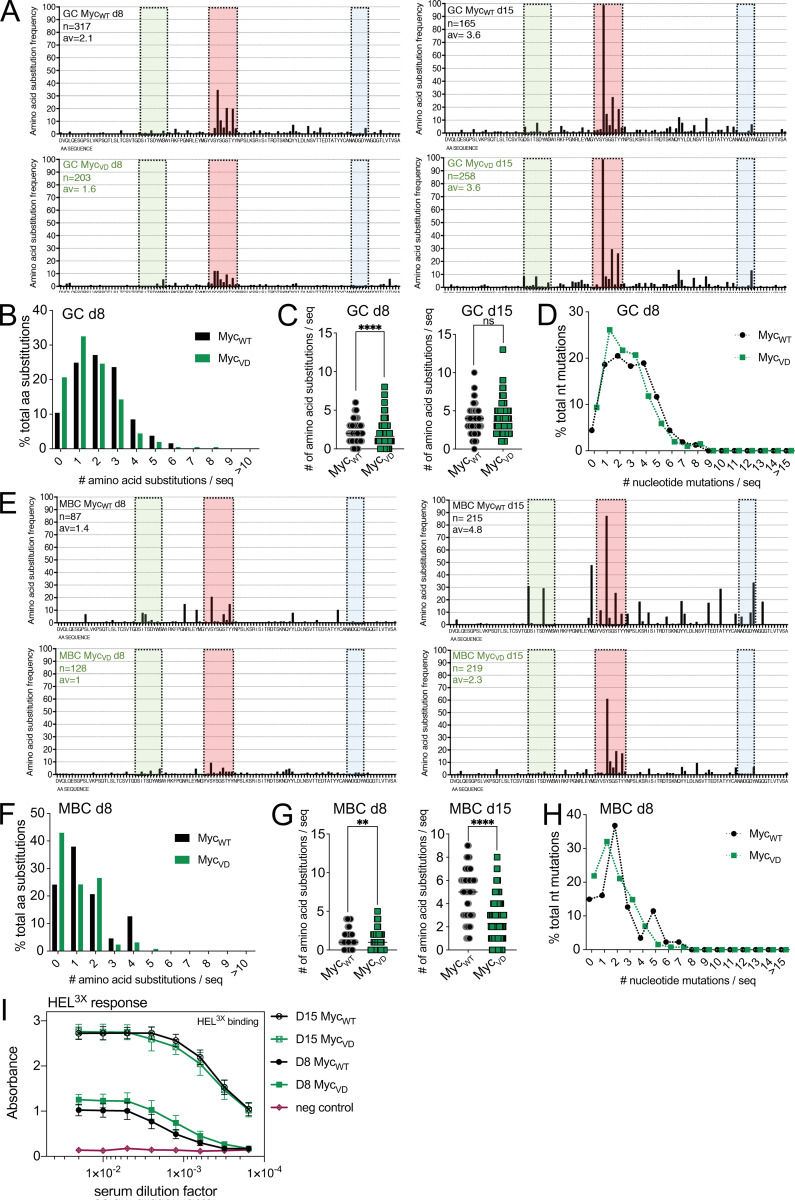
**Affinity maturation in the absence of MYC–MIZ1 complexes. (A)** Quantification of amino acid substitutions in the IgH V-region of SWHEL in IgG1^+^ GC B cells at day 8 (two left panels) and day 15 (two right panels) after HEL^3X^ immunization of recipient mice transferred with Myc_WT_SWHEL and Myc_VD_SWHEL B cells. **(B)** Frequency of GC B cells carrying the specified number of amino acid substitutions, analyzed at day 8 after HEL^3X^ immunization. **(C)** Average amino acid substitutions frequency across the IgH V-region of SWHEL in IgG1^+^ GC B cells at day 8 and day 15 after HEL^3X^ immunization of recipient mice transferred with Myc_WT_SWHEL and Myc_VD_SWHEL B cells. **(D)** Frequency of nucleotide substitutions across the IgH V-region of SWHEL within IgG1^+^ GC B cells at day 8 after HEL^3X^ immunization. **(E)** Quantification of the amino acid substitutions in the IgH V-region of SWHEL in IgG1^+^ CD273^+^ MBCs at day 8 (two left panels) and day 15 (two right panels) after HEL^3X^ immunization of recipient mice transferred with Myc_WT_SWHEL and Myc_VD_SWHEL B cells. **(F)** Frequency of IgG1^+^ CD273^+^ MBCs carrying the specified number of amino acid substitutions, analyzed at day 8 after HEL^3X^ immunization. **(G)** Average amino acid substitutions frequency across the IgH V-region of SWHEL in IgG1^+^ CD273^+^ MBCs at days 8 and 15 after HEL^3X^ immunization of recipient mice transferred with Myc_WT_SWHEL and Myc_VD_SWHEL B cells. **(H)** Frequency of nucleotide substitutions across the IgH V-region of SWHEL within IgG1^+^ CD273^+^ MBCs at day 8 after HEL^3X^ immunization. **(I)** ELISA of serum of recipient mice transferred with Myc_WT_SWHEL and Myc_VD_SWHEL B cells to determine affinity maturation of IgG1 antibody response to HEL^3X^ at days 8 and 15 after HEL^3X^ immunization. Each symbol (C and G) represents an individual sequence; small horizontal lines show the mean. Values represent mean and SEM (I). Cumulative analysis of five mice per genotype per time point. **, P ≤ 0.01; ****, P ≤ 0.0001 (unpaired two-tailed Student’s *t* test). Green, CDR1; red, CDR2; blue, CDR3. av, average amino acid substitution per sequence; n, number of analyzed sequences.

To determine the BCR somatic mutation pattern and affinity maturation to HEL^3X^, we FACS-purified GC cells and MBCs at day 8 and 15 after HEL^3X^ immunization, followed by cloning and sequencing of the IgH variable region. The replacement of a tyrosine at position 53 to aspartic acid in SWHEL IgH (Y53D) leads to ∼100-fold increased affinity for HEL^3X^ (Phan et al., 2003). At day 8 after HEL^3X^ immunization, ∼9% of Myc_VD_SWHEL GC B cells had the Y53D amino acid change compared with ∼24% of Myc_WT_SWHEL cells (Fig. 7 E and Fig. S4 A). The average number of amino acid substitutions was also reduced in Myc_VD_SWHEL GC B cells (average, 1.6/sequence) compared with Myc_WT_SWHEL (average, 2.1/sequence; Fig. S4, A–C). However, the number of nucleotide mutations in the SWHEL IgH of GC B cells was similar between genotypes (Fig. S4 D), suggesting that somatic hypermutation per se was not impaired. The observed differences in GC B cells at day 8 were nevertheless transient given that at day 15 after HEL^3X^ immunization no significant differences were found between genotypes (Fig. 7, E–G; and Fig. S4, A–C). MBCs at day 8 after HEL^3X^ immunization of either genotype did not display Y53D mutations (Fig. 7 H and Fig. S4 E). However, the average number of amino acid substitutions was lower in Myc_VD_SWHEL MBCs (average, 1/sequence) compared with Myc_WT_SWHEL (average, 1.4/sequence; Fig. S4, E–G). Still, only small differences were found with respect to nucleotide mutations, with ∼80% of Myc_VD_SWHEL MBCs carrying a somatically mutated BCR compared with ∼85% of Myc_WT_SWHEL MBCs (Fig. S4 H). At day 15 of analysis, the absence of MYC–MIZ1 complexes significantly impacted BCR antigen affinity of MBCs, with ∼60% of Myc_VD_SWHEL MBCs cells carrying Y53D mutations compared with ∼87% of Myc_WT_SWHEL cells (Fig. 7 H and Fig. S4 E). This was accompanied by a reduced number of amino acid substitutions in Myc_VD_SWHEL MBCs (average, 2.3/sequence) compared with Myc_WT_SWHEL control (average, 4.8/sequence; Fig. 7 I; and Fig. S4, E–G). Nevertheless, the vast majority of Myc_VD_SWHEL MBCs (∼90%) displayed a somatically mutated BCR (Fig. 7 J), further demonstrating that the observed increase in MBC differentiation when MYC–MIZ1 complexes are absent occurs primarily through a GC-dependent path. To investigate if the absence of MYC–MIZ1 complexes altered antigen affinity of the PC pool, we evaluated the binding of IgG1 antibodies in the serum of mice to HEL^3X^ at days 8 and 15 after immunization (Fig. S4 I). No significant differences were found between genotypes, indicating that the absence of MYC–MIZ1 complexes primarily impacts the antigen affinity of the MBC pool. In summary, the absence of MYC–MIZ1 complexes had a lasting impact in the MBC pool by increasing its size and decreasing its affinity for the antigen.

### MYC–MIZ1 complexes restrict MBC differentiation when affinity-based selection is absent

**Figure 8.**
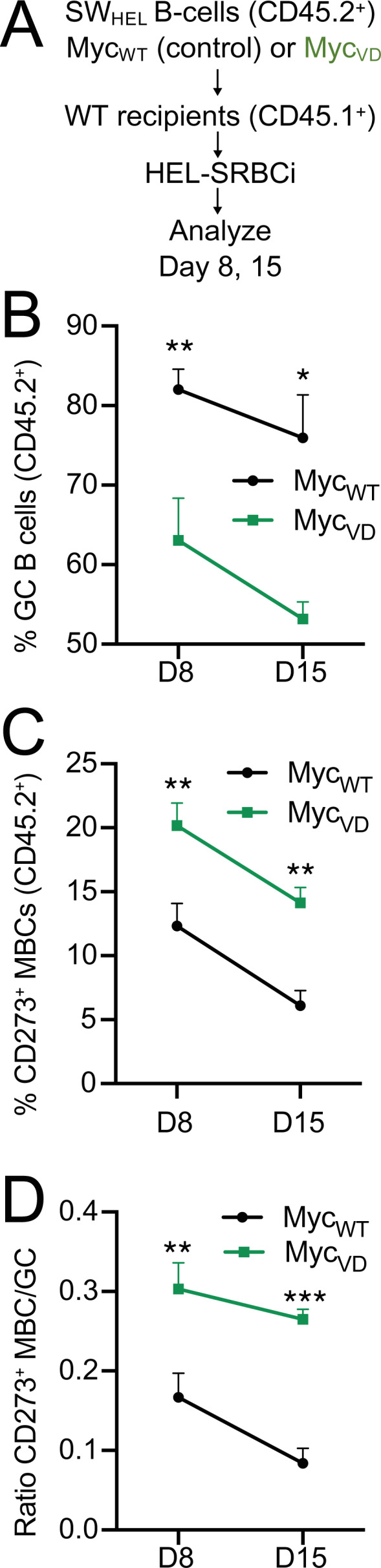
**MYC–MIZ1 complexes restrict MBC differentiation in the absence of affinity-based selection. (A)** Experimental design. **(B)** Kinetics of GC B cells within splenic CD45.2^+^ donor cells at days 8 and 15 after HEL immunization. **(C)** Kinetics of CD273^+^ MBCs within splenic CD45.2^+^ donor cells at days 8 and 15 after HEL immunization. **(D)** Ratio of CD273^+^ MBCs to GC B cells at days 8 and 15 after HEL immunization. **(B–D)** Mean and SEM. *, P ≤ 0.05; **, P ≤ 0.01; ***, P ≤ 0.001 (unpaired two-tailed Student’s *t* test). Data are representative of three (B–D) independent experiments and of the cumulative analysis of five mice per genotype per time point.

**Figure S5.**
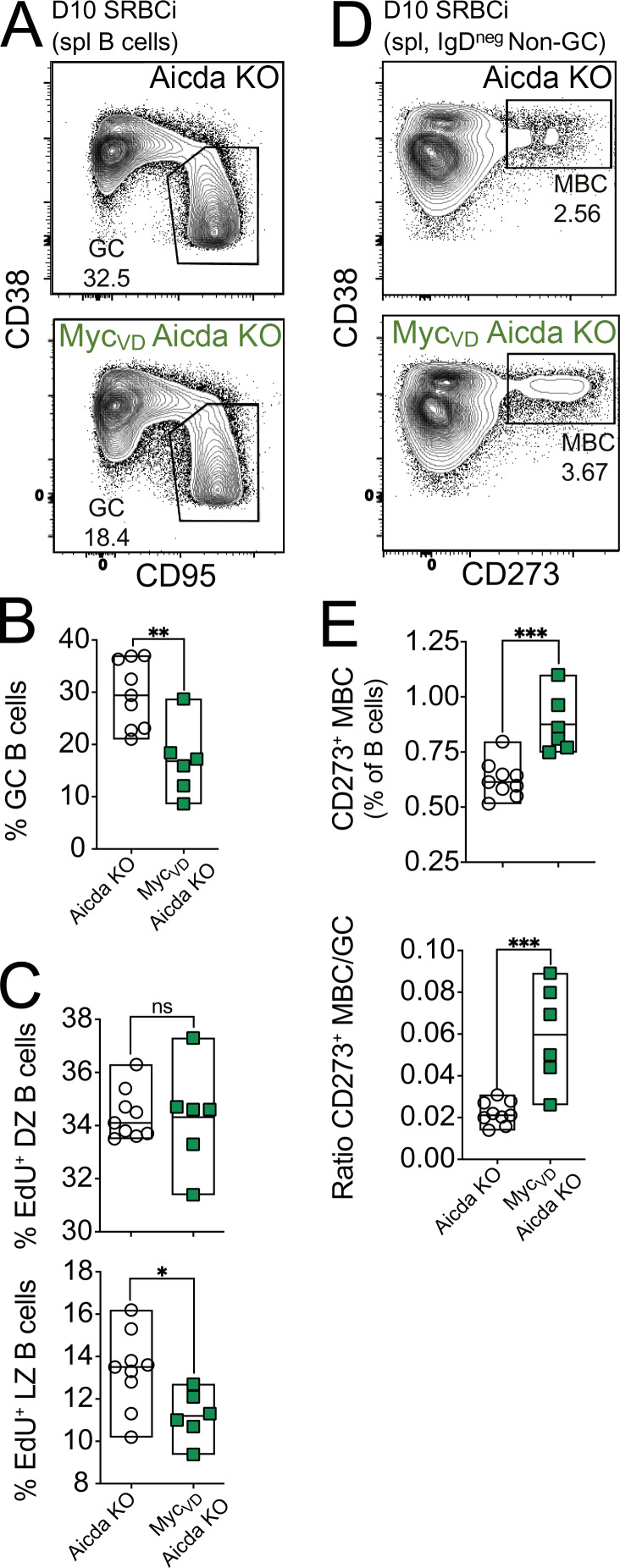
**MYC–MIZ1 complexes restrict MBC differentiation in the absence *Aicda*. (A)** Representative flow cytometry of splenic GC B cells of Aicda KO and Myc_VD_ Aicda KO at day 10 after SRBC immunization (SRBCi). spl, spleen. **(B)** Cumulative data for fraction of splenic GC B cells in Aicda KO and Myc_VD_ Aicda KO at day 10 after SRBC immunization. **(C)** Cumulative data of EdU incorporation in splenic DZ B cells (top) and LZ B cells (bottom) in Aicda KO and Myc_VD_ Aicda KO at day 10 after SRBC immunization. **(D)** Representative flow cytometry gating strategy for splenic CD273^+^ MBCs of Aicda KO and Myc_VD_ Aicda KO at day 10 after SRBC immunization (SRBCi). spl, spleen. **(E)** Cumulative data of splenic CD273^+^ MBCs in Aicda KO and Myc_VD_ Aicda KO at day 10 after SRBC immunization. Top: Fraction of cells. Bottom: ratio of MBC to GC. Each symbol (B, C, and E: Myc_WT_ *n* = 9, Myc_VD_ *n* = 6) represents represent an individual mouse; small horizontal lines indicate mean. *, P ≤ 0.05; **, P ≤ 0.01; ***, P ≤ 0.001 (unpaired two-tailed Student’s *t* test). Data are representative of three independent experiments. ns, not significant.

LZ B cells bearing BCRs with lower antigen affinity are favored to differentiate into MBCs (Shinnakasu et al., 2016). Therefore, it was possible that increased MBC differentiation in Myc_VD_ was primarily the consequence of the transitory delay in GC B cell affinity maturation in these mice. To test this hypothesis, we used the SWHEL system and immunized recipient mice with HEL, for which BCR affinity is very high (2 × 10^10^ M^−1^; Paus et al., 2006), effectively curtailing antigen affinity–based selection (Fig. 8 A). Similar to the results using HEL^3X^ immunization (Fig. 7, A–D), the fraction of GC B cells was reduced within Myc_VD_SWHEL B cells, whereas the fraction of CD273^+^ MBCs was increased, compared with Myc_WT_SWHEL at all time points of analysis (days 8 and 15; Fig. 8, B–D). We also performed an experiment using mice deficient for the enzyme *Aicda* that is critically required for somatic hypermutation and class-switch recombination (Muramatsu et al., 2000). *Aicda*-deficient Myc_VD_, similarly to Myc_VD_ (Fig. 3), displayed a significant reduction in the fraction of GC B cells compared with mice with *Aicda* deficiency alone (Fig. S5, A and B). We also observed that LZ B cells of *Aicda*-deficient Myc_VD_ were impaired in cell cycle engagement compared with *Aicda*-deficient mice, whereas the fraction of DZ B cells engaged in cell cycle was not different between genotypes (Fig. S5 C). Lastly, *Aicda*-deficient Myc_VD_ displayed a significantly increased fraction of MBCs compared with mice with *Aicda* deficiency alone (Fig. S5, D and E). We concluded that MYC–MIZ1 complexes in LZ MYC^+^ cells restricted MBC differentiation even when affinity-based selection was absent.

## Discussion

MBCs are key for long-term protection from reinfection. However, the mechanisms underlying MBC differentiation are unclear. Here, we found that LZ MYC^+^ cells mostly coexpressed the transcription activators MYC and MIZ1. Complexes formed by MYC and MIZ1 in these cells were required for repression of MIZ1 target genes, effective cell cycle engagement of LZ B cells, GC expansion, and PC formation. Notably, most of the MIZ1 target genes repressed by MYC–MIZ1 complexes were enriched in MBCs, and the absence of MYC–MIZ1 complexes increased MBC differentiation. Whether these events are interdependent or independent, the first suggesting that restriction of MBC differentiation in LZ MYC^+^ cells is required for effective GC expansion and PC formation, remains to be determined. The signals driving MIZ1 expression in LZ MYC^+^ cells also need further investigation, because in contrast to MYC (Luo et al., 2018), ex vivo BCR and CD40 coengagement was not sufficient to induce MIZ1 in GC B cells. Further, the discrepancy between *Miz1* gene and protein expression argues for a tight regulation at the posttranscriptional level. HUWE1 is an E3 ubiquitin ligase that ubiquitinates MIZ1, triggering its proteasomal degradation (Yang et al., 2010). However, previous analysis of HUWE1-deficient mice suggest a limited function for this E3 ubiquitin ligase in the GC reaction (Hao et al., 2012).

MYC–MIZ1 complexes were not equally required in all LZ MYC^+^ cells, given that apparently a fraction entered cell cycle normally. These data and of others (Ise et al., 2018) highlight the need to investigate populational diversity within LZ MYC^+^ cells. We also observed that cellular proliferation of activated B cells in vitro was mostly unimpaired. Thus it is possible that MYC–MIZ1 complexes are primarily required at specific B cell stages and/or to counteract autocrine and/or paracrine (i.e., microenvironmental) antiproliferative signals (van Riggelen et al., 2010).

Most of the identified MIZ1 target genes up-regulated in the absence of MYC–MIZ1 complexes were enriched in MBCs. Although for many a function in B cells is unknown, for others (namely *Arhgef1*, *Gadd45g*, *Sh2b3*, and *Map2k7*), tumor-suppressive and/or antiproliferative activity was described (Lu et al., 2001; Lv et al., 2017; Muppidi et al., 2014; Perez-Garcia et al., 2013; Thalheimer et al., 2014). This knowledge is in agreement with work demonstrating that quiescent B cells in the LZ are enriched for MBC precursors (Laidlaw et al., 2017; Suan et al., 2017; Wang et al., 2017). Thus, MIZ1 transcriptional activity may promote a quiescent state, which in turn permits MBC differentiation. Such activity is restrained by MYC–MIZ1 complexes in LZ MYC^+^ cells. Whether MIZ1 transcriptional activity is itself required for MBC differentiation needs further investigation.

The finding that MIZ1 targets genes with B cell tumor suppressor and/or antiproliferative function may be relevant for GC B cell lymphomagenesis. It is tempting to speculate that interference with MBC differentiation could be oncogenic, similar to what we and others demonstrated for PC differentiation (Calado et al., 2010; Mandelbaum et al., 2010; Zhang et al., 2015).

In cancer, *Cdkn1a* is a well-characterized target of MYC–MIZ1 complexes (Herold et al., 2002; Walz et al., 2014). However, *Cdkn1a* expression was unchanged in LZ B cells in the absence of MYC–MIZ1 complexes. Previous work showed that BCL6 can form a transcriptional repressor complex with MIZ1 that represses *CDKN1A* expression in GC-derived lymphoma cell lines (Basso and Dalla-Favera, 2010; Phan et al., 2005). Also, others have also shown that EZH2 is required in GC B cells for repression of *Cdkn1a* expression (Béguelin et al., 2017). Thus, it is possible that redundancy in the regulation of *Cdkn1a* expression exists in GC B cells. In contrast, we found increased expression of *Cdkn1b* in the absence of MYC–MIZ1 complexes. However, and although our analysis and that of others have not identified *Cdkn1b* as a direct MIZ1 target (Walz et al., 2014), this has been suggested by other studies (Basu et al., 2009; Yang et al., 2001), urging further investigation.

LZ B cells with high *Bach2* expression are favored to differentiate into MBCs (Shinnakasu et al., 2016). However, *Bach2* expression was not altered in the absence of MYC–MIZ1 complexes. *Bach2* expression is inversely correlated to the strength of T cell help (Shinnakasu et al., 2016). On the contrary, *Myc* is induced upon positive selection downstream of T cell help (Luo et al., 2018), and MYC levels were not altered by the absence of MYC–MIZ1 complexes, suggesting that T cell help was not affected. As a consequence, changes in *Bach2* expression levels would not be expected in the absence of MYC–MIZ1 complexes. This does not contradict the role of *Bach2* in the regulation of MBC differentiation (Shinnakasu et al., 2016). First, *Bach2* may be required for MBC differentiation of a different LZ B cell subset; second, we found that MYC–MIZ1 complexes repressed the expression of the gene encoding the MAFK transcription factor, with which BACH2 is bound at most target genes (Huang et al., 2014; Oyake et al., 1996). Thus, given that strong T cell help reduces *Bach2* expression but does not extinguish it (Shinnakasu et al., 2016), increased *Mafk* expression in the absence of MYC–MIZ1 complexes could favor MBC differentiation.

Increased MBC differentiation, together with impaired GC B cell expansion, was also found when LZ B cells cannot sense IL21 and when LZ to DZ migration is abrogated by impaired CXCR4 signaling (Bannard et al., 2013; Barinov et al., 2017; Linterman et al., 2010; Zotos et al., 2010). In both scenarios, antigen affinity was reduced, possibly favoring MBC differentiation (Shinnakasu et al., 2016; Weisel et al., 2016). The absence of MYC–MIZ1 complexes led only to a transient delay in GC B cell affinity. Thus, and although we cannot exclude a contribution of this phenomenon toward increased MBC differentiation, we found that MYC–MIZ1 complexes restricted MBC differentiation even when affinity-based selection was absent. The MBC phenotype in IL21/IL21R deficiency was attributed to a GC-independent path given that most MBCs had unmutated BCRs, whereas for CXCR4 deficiency, the involvement of GCs was suggested (Bannard et al., 2013; Linterman et al., 2010; Zotos et al., 2010). The latter conclusion was based on the detection of CD73 expression and MBC BrdU incorporation in pulse-chase experiments (Bannard et al., 2013). We found that increased MBC differentiation in the absence of MYC–MIZ1 complexes occurred primarily through a GC-dependent path: (1) MYC–MIZ1 complexes were required for GC expansion rather than formation; (2) an increased number of EdU^+^ MBCs was formed during critical time points of GC expansion; (3) the fraction of IgG1^+^ CD73^+^ MBCs and LZ MBC precursors (CCR6^+^ LZ B cells) was increased in Myc_VD_ compared with Myc_WT_; and (4) the vast majority (∼90%) of MBCs in Myc_VD_ display somatically mutated BCRs.

Our work uncovered that MYC–MIZ1 complexes in LZ MYC^+^ cells are required for effective GC expansion and PC formation and to restrict MBC differentiation. Until now, a physiological function for MYC–MIZ1 complexes was unknown (Wiese et al., 2013). MIZ1, similarly to MYC, is required for early B and T cell development; however, these functions were shown to be independent of MYC–MIZ1 complexes (Douglas et al., 2001; Kosan et al., 2010; Saba et al., 2011; Vallespinós et al., 2011). MIZ1 homologues and the conservation of a valine in position 394 in MYC, critical for the interaction with MIZ1, are found only in vertebrates (Conacci-Sorrell et al., 2014). Thus, compared with other MYC network members, like MAX and MXD proteins, MYC–MIZ1 complexes are a late addition in evolution (Conacci-Sorrell et al., 2014). Interestingly, such evolutionary timeframe is similar to that of AICDA (Conticello et al., 2005).

MYC–MIZ1 complexes per se are not essential for life (Wiese et al., 2013). As a consequence, this protein complex is a viable candidate for intervention in cancer and vaccination. With respect to the latter, an increased MBC pool size with lower affinity for the primary immunizing antigen, as observed in the absence of MYC–MIZ1 complexes, may permit the recognition of similar but different antigens and further affinity maturation (Bannard and Cyster, 2017; Inoue et al., 2018; McHeyzer-Williams et al., 2015; Mesin et al., 2016; Takahashi and Kelsoe, 2017; Tas et al., 2016). This could be important in vaccination for protection against pathogenic substrains and evolved mutants (Inoue et al., 2018; Victora and Wilson, 2015). Thus, interventions that modulate the activity of MYC–MIZ1 complexes may tailor the GC response to meet individual humoral memory requirements for infection control and prevention.

## Materials and methods

### Mice

Myc_VD_, Cdkn1a KO, AID-Cre-ERT2, and the transgenic SWHEL allelic BCR system mouse strains have been previously described (Brugarolas et al., 1995; Calado et al., 2012; Casola et al., 2006; de Alboran et al., 2001; Dogan et al., 2009; Phan et al., 2003; Saba et al., 2011). Mice were maintained on the C57BL/6 background and bred at the Francis Crick Institute biological resources facility under specific pathogen–free conditions. Animal experiments were performed in accordance with national and institutional guidelines for animal care and approved by The Francis Crick Institute biological resources facility strategic oversight committee (incorporating the Animal Welfare and Ethical Review Body) and by the Home Office, UK.

### Immunization, adoptive transfers, and in vivo treatments

For T cell–dependent immunization, 8- to 12-wk-old mice were injected i.v. with 10^9^ defibrinated SRBCs (TCS Bioscience) in PBS. EdU (Invitrogen) was dissolved in sterile PBS (5 mg/ml); for proliferation studies, 1 mg in a volume of 200 µl was injected i.p. 3 h before analysis; for the assessment of the kinetics of the formation of MBCs, 1.5 mg in a volume of 300 µl was injected i.p. every 12 h for 3 d (protocol adapted from Weisel et al., 2016). Adoptive transfers were performed into CD45.1^+^ or CD45.1^+^/CD45.2^+^ congenic mice. Briefly, 3 × 10^4^ HEL-binding B cells were injected i.v. into congenic recipients, followed by i.v. immunization the next day with 2 × 10^8^ SRBCs conjugated to a specific recombinant HEL protein. SRBCs in Alsever’s (TCS Bioscience) were conjugated to recombinant HEL (Sigma) or HEL^3X^ (R. Brink) with 1-ethyl-3-(3-dimethylaminopropyl) carbodiimide hydrochloride (Sigma) as previously described (Goodnow et al., 1988). CD45.2^+^ splenic B cells from SWHEL donor mice were purified by CD43 (Ly-48) MicroBeads (Miltenyi Biotec) depletion. For analyses and cell FACS sorting, CD45.2^+^ donor splenocytes were enriched by CD45.1-negative selection using anti-mouse biotinylated CD45.1 antibody (clone A20; eBioscience) and Anti-Biotin MicroBeads (Miltenyi Biotec).

### Histology, immunohistochemistry, and immunofluorescence

Spleens were fixed with 10% neutral buffered formalin (Thermo Fisher) and embedded in paraffin. Sections were stained with hematoxylin (Sigma) and biotinylated peanut agglutinin (Vector). Images were acquired with Zeiss Axio Scan.Z1 Slide Scanner and visualized with Photoshop (v12.1; Adobe). ImageJ (v2.0.0) was used to quantify GC B cell area and number of GCs per spleen section. For immunofluorescence, spleens were embedded in optimum cutting temperature compound (Sakura) and flash frozen in liquid nitrogen. 8- to 30-µM tissue sections were cut on an OTF5000 cryostat (Bright Instruments), fixed in 4% paraformaldehyde (Thermo Fisher), permeabilized and blocked in PBS with 0.3% Triton X-100 (Sigma) and 10% normal goat serum (Sigma). Samples were stained with antibodies in PBS containing 0.3% Triton X-100 with 1% bovine serum albumin (Thermo Fisher). Every incubation was followed by three washes with PBS. To prevent cross-reactivity, samples were blocked with PBS containing 10% normal rabbit serum (Sigma). Images were acquired with a Leica Sp5 confocal microscope, using sequential acquisition between frames with 405-, 488-, 555-, and 647-nm laser excitations. Images were analyzed with Imaris software (Bitplane); cells were automatically identified by the software based on nuclear Hoechst 33342 staining.

### Antibodies

The following antibodies were used: rabbit anti-mouse caspase 3, clone C92-605 (BD Biosciences); rat anti-mouse CD19, clone 6D5 (BioLegend); rat anti-mouse CD23, clone B3B4 (BioLegend); rat anti-mouse CD35, clone 8C12 (BD Biosciences); rat anti-mouse CD38, clone 90 (BioLegend); rat anti-mouse CD45R/B220, clone RA3-6B2 (BioLegend); mouse anti-mouse CD45.1, clone A20 (eBioscience); mouse anti-mouse CD45.2, clone 104 (BD Biosciences); rat anti-mouse CD73, clone TY/23 (BD Biosciences); rat anti-mouse CD86, clone GL1 (BioLegend); rat anti-mouse CD93 (AA.1), clone AA4.1 (eBioscience); Armenian hamster anti-mouse CD95 (FAS), clone Jo2 (BD Biosciences); rat anti-mouse CD138 (Syndecan-1), clone 281–2 (BioLegend); rat anti-mouse CD184 (CXCR4), clone 2B11 (eBioscience); rat anti-mouse CD273 (PD-L2), clone TY25 (BioLegend); rat anti-mouse IgD, clone 11-26c.2a (BD Biosciences); rat anti-mouse IgG1, clone A85-1 (BD Biosciences); rat anti-mouse IgM, clone II/41 (BD Biosciences); Armenian hamster anti-mouse PD1(CD279), clone J43 (eBioscience); rat anti-mouse CXCR5(CD185), clone (SPRCL5); rat anti-mouse CD4, Clone (GK1.5); Armenian hamster anti-mouse CD3, clone (145-2C11); Armenian hamster anti-mouse CD80(B7-1), clone (16-10A1); rat anti-mouse CD21/CD35(CR2/CR1), clone (7E9); mouse anti-HEL, clone HyHEL9 (R. Brink); rabbit anti-Myc N-262 (Santa Cruz); rabbit anti-Myc, clone Y69 (Abcam); rabbit anti-Zbtb17 (Sigma); goat anti-rabbit IgG (H+L; Molecular Probes), fluorochrome-labeled Streptavidin (BioLegend, eBioscience, and BD Biosciences); biotinylated peanut agglutinin (Vector); and IgG from rabbit serum (Sigma).

### In vitro cell stimulation and transduction

Splenic naive B cells were purified by CD43 depletion (Miltenyi). GC B cells were purified as previously described (Luo et al., 2018). Naive and GC B cells were cultured in vitro in the presence of 10 µg/ml (anti-IgM + anti-IgG; Jackson ImmunoResearch) and/or 20 µg/ml anti-CD40 antibody (FGK45; Bio X Cell) with or without 25 ng/ml IL-4. For cell proliferation studies, naive B cells were stained with CellTrace Violet (Thermo Fisher) according to the manufacturer’s protocol. HEK293T cells were transiently transduced with a MIZ1-IRES-GFP expressing vector or GFP control (VectorBuilder) using Lipofectamine (Invitrogen). Cells were analyzed 48 h after transduction.

### Flow cytometry and ELISA

Single-cell suspensions of spleen were prepared in FACS buffer (2% FBS and 2 mM EDTA, in PBS; Gibco) and treated with Gey’s solution for erythrocyte lysis. Single-cell suspensions were stained with antibodies. The use of biotinylated antibodies was followed by incubation with fluorochrome labeled streptavidin (1/200 dilution). For the analyses of SWHEL mice, HEL-binding cells were stained with 50 ng/ml HEL (Sigma) followed by HyHEL9-A647 (R. Brink). Dead cells were excluded using Zombie NIR Fixable Viability Kit (BioLegend). For detection of EdU incorporation, cells were fixed for 15 min at room temperature in 4% paraformaldehyde (Thermo Fisher) after surface marker and viability dye staining. Fixation was followed by Click-iT EdU A647 flow cytometry assay kit or Click-iT Plus EdU Alexa Fluor 647 flow cytometry assay kit (Life Technologies) as indicated by supplier. For the detection of cleaved caspase-3, samples were fixed for 15 min at room temperature in 4% paraformaldehyde (Thermo Fisher) after surface marker and viability dye staining, followed by intracellular staining with BD Cytofix/Cytoperm staining kit (BD Biosciences), as per manufacturer’s specifications. The detection of the transcription factor was performed after surface marker and viability dye staining with True-Nuclear Transcription Factor Buffer Set (BioLegend), following supplier’s instructions. To prevent cross-reactivity, samples were blocked with 10% normal rabbit serum. Samples were acquired on an LSR-Fortessa (BD Biosciences) with FACS-Diva software (BD Biosciences), and data were analyzed with FlowJo software (v10.3; Tree Star). MBCs were FACS purified using the following antibody panel: CD273^+^CD138^neg^ B220^+^ CD19^+^ CD38^high^ IgD^low^. Cell populations were defined by the following surface markers: LZ GC B cells (B220^+^ CD19^+^ CD38^low^ CD95^high^ CXCR4^low^ CD86^high^), DZ GC B cells (B220^+^ CD19^+^ CD38^low^ CD95^high^ CXCR4^high^ CD86^low^), PCs (CD19^low^ CD138^+^), and IgM^+^ and IgG1^+^ MBCs (B220^+^ CD19^+^ CD38^high^ IgD^low^ CD273^+^); FO B cells (AA4.1^neg^ B220^+^ CD19^+^ CD38^high^ IgD^+^ CD21^high^ CD23^high^). Anti-HEL^3X^ IgG1 antibodies in sera were analyzed by ELISA as previously described (Phan et al., 2003, 2006).

### IgH somatic mutation analysis

IgG1^+^ CD273^+^ HEL^+^ MBCs and IgG1^+^ HEL^+^ GC B cells from SWHEL mice were sorted using a FACSAria III or FACSAria Fusion (BD Biosciences). Cells were sorted into proteinase K (Qiagen) containing buffer, as previously described (Brink et al., 2015; Paus et al., 2006). The SWHEL Ig heavy-chain variable region exon was PCR amplified using Taq DNA polymerase (Thermo Fisher) with the primers 5′-GTT​GTA​GCC​TAA​AAG​ATG​ATG​GTG-3′ and 5′-GAT​AAT​CTG​TCC​TAA​AGG​CTC​TGA​G-3′. The primary PCR product was further amplified with the nested primers 5′-TTG​TAG​CCT​AAA​AGA​TGA​TGG​TGT​TAA​GTC-3′ and 5′-CAA​CTT​CTC​TCA​GCC​GGC​TC-3′. Nested PCR product was isolated using QIAquick Kit (Qiagen) and cloned into pGEM-T Easy vector (Promega), as per the manufacturer’s instructions. DH5α-competent bacteria (Life Technologies) were transformed and single colonies were grown in 96 deep-well culture blocks (Macherey-Nagel). DNA was extracted automatically with Biorobot Universal System (Qiagen) using Nucleospin 96 Plasmid kits (Macherey-Nagel) and Sanger-sequenced with T7, SP6, M13 forward, and/or M13 reverse primers. Sequenced DNA was run against VBASE2 database (Retter et al., 2005). Using HyHEL10 sequence as a reference, mature VDJ DNA sequences were analyzed for the presence of somatic mutation events, and the translated protein sequences were examined for amino acid substitutions.

### Gene expression analysis and ChIP-seq

For the gene expression profiling of Myc_WT_ and Myc_VD_ LZ GC B cells (CD138^neg^, B220^+^, CD19^+^ CD38^low^, CD95^high^, CXCR4^low^ CD86^high^), Myc_WT_ MBCs (CD138^neg^, B220^+^, CD19^+^, AA4.1^neg^, CD38^+^, IgD^neg^, CD273^+^), and Myc_WT_ FO B cells (AA4.1^neg^, B220^+^, CD19^+^, CD38^high^, IgD^+^, CD21^high^, CD23^high^) were sorted by flow cytometry at day 10 after immunization with SRBCs using a FACSAria III or a FACSAria Fusion (BD Biosciences). Cells were sorted in RTL Buffer Plus (Qiagen) containing 1% β-mercaptoethanol (Sigma) and RNA purified using AllPrep DNA/RNA Mini and Micro Kits (Qiagen) following the manufacturer’s instructions. RNA-seq was performed at the Francis Crick Institute advanced sequencing facility. The RSEM package (v1.3.0; Li and Dewey, 2011) and STAR (v2.5.2a; Dobin et al., 2013) were used to align reads to the mouse mm10 transcriptome, taken from Ensembl (vGRCm38) and available at the University of California, Santa Cruz (https://genome.ucsc.edu). For RSEM, all parameters were run as default using the “–forward-prob 0” option for a strand-specific protocol. Differential expression analysis was performed with DESeq2 package (v.1.12.4; Love et al., 2014) within R (v3.3.1; R Core Team, 2008; http://www.R-project.org). Genes were considered to be differential expressed if P < 0.05. GSEA was performed using GSEA software (v2.2.3) from the Broad Institute (Subramanian et al., 2005). All analyses for RNA-seq–generated expression profiles were done with ranked gene lists using Wald statistics. For ChIP, splenic B cells from C57BL/6 mice were purified using anti-mouse CD43 (Ly-48) MicroBeads (Miltenyi Biotec) depletion and treated with Gey’s solution for erythrocyte lysis. Cells (2 × 10^6^ cells/ml) were cultured at 37°C (5% CO_2_) in DMEM supplemented GlutaMAX, nonessential amino acids, penicillin-streptomycin, Hepes (Gibco), β-mercaptoethanol (Sigma), and 10% fetal bovine serum (Thermo Fisher) containing anti-IgM (5 µg/ml), CD40 ligand (0.5 µg/ml), and IL-4 (10 ng/ml) and harvested after 16 h. ChIP protocol was adapted from Seitz et al. (2011). Briefly, cells in B cell media were fixed with formaldehyde (Sigma) at final concentration of 1% for 10 min at room temperature. Glycine (Thermo Fisher) was added at a final concentration of 0.125 M for 5 min at room temperature. Cells were washed, pelleted, and stored at −80°C. After cell lysis, the DNA of 35 × 10^6^ cells was fragmented using BIORUPTOR 200 immersion sonicator under the following conditions: high power, 30 s on, 30 s off (40 cycles). 100 μl of Pierce Protein A/G Magnetic Beads were coupled to 10 µg of antibody or control rabbit IgG antibody (Sigma). DNA was incubated with antibody-coupled beads, and after uncoupling, DNA was purified with NucleoSpin Gel and PCR Clean-up (Macheray-Nagel) following the manufacturer’s protocol for SDS-rich samples using Buffer NTB (Macheray-Nagel). Samples were sequenced on an Illumina HiSeq2500 generating 100bp single ended reads. ChIP-seq reads were aligned to the mouse mm10 genome assembly using BWA version 0.7.15 (Li and Durbin, 2010) with a maximum mismatch of two bases. Picard tools version 2.1.1 (http://www.broadinstitute.github.io/picard) was used to sort, mark duplicates, and index the resulting alignment bam files. Files were normalized, and tdf files (for visualization purposes) were created using IGVtools version 2.3.75 software (http://www.broadinstitute.org/igv) by extending reads by 50 bp and normalizing to 10 million mapped reads per sample. Peaks were called using the standard parameters by comparing immunoprecipitated samples to their respective input and/or IgG controls using MACS version 2.1.1 (Zhang et al., 2008). Peaks called by MACS were annotated using the “annotatepeaks” function in the Homer version 4.8 software package (Heinz et al., 2010). Common and unique peaks across experiments were determined using a custom script. Datasets are available at the National Center for Biotechnology Information Gene Expression Omnibus under the following accession numbers: GSE129262, GSE80669, GSE77319, GSE4142, GSE76502, GSE11961, and GSE98419.

### Quantification and statistical analysis

Data were analyzed with unpaired two-tailed Student’s *t* test; a P value of ≤0.05 was considered significant. Prism (v7 and v8, GraphPad) was used for statistical analysis. A single asterisk (*) in the graphs of figures represents a P value ≤0.05, double asterisks (**) a P value ≤0.01, triple asterisks (***) a P value ≤0.001, and quadruple asterisks (****) a P value ≤0.0001; “ns” stands for not statistically significant (i.e., a P value >0.05). Data in text and figures are presented as median or mean ± SEM; each case is indicated in the figure legends.

### Online supplemental material

Fig. S1 shows MYC and MIZ1 expression in GC B cell populations, the identified MYC–MIZ1 complex target genes, and PC and CD4^+^ CXCR5^+^ PD1^+^ T cell populations in the absence of MYC–MIZ1 complexes. Fig. S2 shows the gene expression of cell cycle–related genes in the absence of MYC–MIZ1 complexes, the characterization of MYC, MIZ1 in activated non-GC B cells, their cell proliferation in the absence of MYC–MIZ1 complexes, and MYC and MIZ1 expression in (pre-)GC B cells. Fig. S3 shows the enrichment for genes up-regulated in MBCs in the absence of MYC–MIZ1 complexes and MBC populations. Fig. S4 shows GC and MBC affinity in the absence of MYC–MIZ1 complexes. Fig. S5 shows that MYC–MIZ1 complexes restrict MBC differentiation in the absence of *Aicda*.
